# *In vivo* evidence that eIF3 stays bound to ribosomes elongating and terminating on short upstream ORFs to promote reinitiation

**DOI:** 10.1093/nar/gkx049

**Published:** 2017-01-24

**Authors:** Mahabub Pasha Mohammad, Vanda Munzarová Pondělíčková, Jakub Zeman, Stanislava Gunišová, Leoš Shivaya Valášek

**Affiliations:** Laboratory of Regulation of Gene Expression, Institute of Microbiology AS CR, Prague, Videnska 1083, 142 20, Czech Republic

## Abstract

Translation reinitiation is a gene-specific translational control mechanism characterized by the ability of some short upstream ORFs to prevent recycling of the post-termination 40S subunit in order to resume scanning for reinitiation downstream. Its efficiency decreases with the increasing uORF length, or by the presence of secondary structures, suggesting that the time taken to translate a uORF is more critical than its length. This led to a hypothesis that some initiation factors needed for reinitiation are preserved on the 80S ribosome during early elongation. Here, using the *GCN4* mRNA containing four short uORFs, we developed a novel *in vivo* RNA–protein Ni^2+^-pull down assay to demonstrate for the first time that one of these initiation factors is eIF3. eIF3 but not eIF2 preferentially associates with RNA segments encompassing two *GCN4* reinitiation-permissive uORFs, uORF1 and uORF2, containing *cis*-acting 5΄ reinitiation-promoting elements (RPEs). We show that the preferred association of eIF3 with these uORFs is dependent on intact RPEs and the eIF3a/TIF32 subunit and sharply declines with the extended length of uORFs. Our data thus imply that eIF3 travels with early elongating ribosomes and that the RPEs interact with eIF3 in order to stabilize the mRNA-eIF3-40S post-termination complex to stimulate efficient reinitiation downstream.

## INTRODUCTION

Gene expression can be regulated at multiple levels including transcription, mRNA processing and localization, protein translation and protein stability. It is now well accepted that regulation at the level of translation makes a significant contribution to the overall regulation of gene expression. On most eukaryotic mRNAs, translation initiation occurs by the scanning mechanism (reviewed in (1)). It begins with the assembly of a 43S pre-initiation complex (PIC), comprising a 40S ribosomal subunit, a ternary complex (TC) composed of eukaryotic initiation factor 2 (eIF2) bound to guanosine triphosphate (GTP) and Met-tRNA_i_^Met^, and eIFs 3, 1 and 1A (reviewed in (2)). The 43S PIC attaches to the eIF4F-bound 5΄ cap structure of mRNA in a process that involves unwinding of its secondary structure by eIFs 4A and 4B forming the 48S PIC. This higher-order complex then scans to the initiation codon where it arrests scanning in an intricate, multi-step process by establishing stable codon–anticodon base-pairing between the initiating AUG and Met-tRNA_i_^Met^. After the start codon recognition, eIF5B promotes joining of a 60S subunit, which results in the ejection of most eIFs including eIF2 and yields elongation-competent 80S ribosomes. However, in some instances, such as it playing a center role in this study, protein synthesis does not begin with canonical initiation but instead results from the so-called reinitiation (REI), which serves as one of many regulatory means of gene expression (reviewed in (3)). Simply speaking, REI is enabled by incomplete recycling of post-termination complexes at the stop codon of an upstream ORF (uORF), in the majority of cases a very short uORF, which allows the small ribosomal subunit to stay mRNA-bound and resume scanning downstream.

uORFs are defined by an initiation codon and an in-frame termination codon separated by at least one additional sense codon. They can overlap with or terminate before the initiation codon of the main protein-coding sequence (CDS). Computational sequence analyses identified uORFs in 13%, 44% and 49% of yeast, mouse and human transcripts, respectively (4,5). Validating these observations, upstream translational initiation sites were recently detected in *>*50% of human transcripts by ribosome profiling (6) and ∼61% of zebrafish uORFs were shown to display signatures of active translation (7). These results suggest a widespread role of uORFs in gene expression regulation. Notably, the presence of multiple uORFs seems to be enriched in certain subgroups of mRNAs, including genes coding for growth factors, transcription factors and other proto-oncogenes (8,9). uORF polymorphism has also been implicated in a variety of human diseases (4,10), and uORF-containing genes are prominent in key cellular processes and functional classes, such as stress response (5), meiosis (11), circadian rhythms (12) and tyrosine kinase activity (13).

According to the scanning model described above, the 43S PIC enters the mRNA at the 5΄ cap and scans sequentially along the 5΄ UTR until it encounters the first AUG codon. Hence, with a few exceptions, uORFs located in the 5΄ UTR of the main ORF will, by default, downregulate its translation, mainly under unstressed conditions (4). In response to cellular stress, however, the presence of uORFs can paradoxically promote increased expression of certain stress-related mRNAs (14), such as for example that of yeast *GCN4*, which is the subject of this work (15). All known structural and functional properties that determine the multitude of regulatory impacts of uORFs on mRNA translation have been recently summarized in a comprehensive uORF literature database (16).

In short, if the scanning ribosome encounters a uORF, it can (i) skip it by the so called leaky scanning mechanism, (ii) translate it and undergo the full recycling phase, (iii) translate it and stall during elongation or termination phases serving as a roadblock or inducing mRNA decay or (iv) translate it, recycle only the large subunit, resume scanning, reacquire the TC and reinitiate further downstream (10,17,18). With a few exceptions, translation REI happens only after translation of a short uORF. It depends on (a) *cis*–acting mRNA features surrounding a given uORF, (b) duration of the uORF elongation, which is determined by the relative length of a short uORF, its sequence and a tendency to form stable secondary structures, (c) a handful of translation initiation factors involved in the first initiation event and (d) the intercistronic distance needed for the acquisition of the new TC (reviewed in (2)). The middle two requirements are united in the long-standing idea that eIFs that are important for promoting reinitiation remain at least transiently associated with the elongating ribosome, and that increasing the uORF length or the ribosome transit time increases the likelihood that these factors are dropped off (19). In this respect, yeast genetic analysis and experiments in mammalian reconstituted systems suggested that eIF3 and eIF4F might be instrumental for this process (20–23). They interact with each other (24,25) and both of them have a favorable location on the solvent-exposed side of the small subunit (2,26–29). Hence, it is conceivable that upon subunit joining, they persistently interact with the post-initiation 40S subunit for a few elongation cycles, somehow ensuring that upon termination only the 60S ribosomal subunit is recycled and the remaining post-termination 40S subunit resumes scanning downstream. However, direct evidence for their involvement in the establishment of the REI competence is still lacking and the molecular details of their REI-promoting role are unclear.

The textbook example of the REI mechanism is the translational control of yeast transcriptional activator *GCN4* (reviewed in (15)), which is governed by four uORFs in the rather intricate fail-safe mechanism (30) (Supplementary Figure S1). This mechanism is very sensitive to the TC levels that are changing in response to different nutrient conditions (31). The first of the four uORFs is efficiently translated under both nutritional replete and deplete conditions, and after its translation, it allows efficient resumption of scanning of the post-termination 40S subunit. The second REI-permissive uORF, uORF2, serves as a backup of uORF1 to capture all ribosomes that eventually leaky scanned the uORF1 AUG (30), especially during stress conditions that increase the frequency of leaky scanning (32–35). In non-stressed cells, where the TC levels are high, nearly all of the rescanning ribosomes can rebind the TC before reaching one of the last two distant uORFs (uORFs 3 and 4), neither of which supports efficient REI; i.e. terminating ribosomes are efficiently recycled and the main *GCN4* ORF is not expressed. Under starvation conditions, the GCN2 kinase phosphorylates eIF2, which suspends formation of new TCs in the cytoplasm. Consequently, post-termination 40S ribosomes traveling from the uORF1 or uORF2 stop codon downstream will require more time to rebind the TC. This will allow a large proportion of them to bypass REI-non-permissive uORF3 and uORF4 and reacquire the TC only past uORF4 but still before the *GCN4* start codon. Thus, whereas the global protein synthesis is significantly down-regulated, protein expression of *GCN4* is concurrently induced.

We and others demonstrated that the high REI competence of uORF1 and uORF2 depends on several *cis*-acting features (15). One of the two most important ones is the AU-rich motif occurring within the first 12 nucleotides (nt) immediately following the uORF1 stop codon (36), and the other is represented by the REI-promoting elements (RPEs) with a specific structural arrangement occurring in the upstream regions of uORF1 and uORF2 (22,30) (Supplementary Figure S2). In detail, uORF1 utilizes four RPEs (i–iv), whereas uORF2 separately utilizes only a single RPE v (similar in sequence with the uORF1-specific RPE i) and, in addition, ‘shares’ RPE ii with uORF1. Besides that, we also implicated the extreme N-terminal domain (NTD) of the a/TIF32 subunit of the eukaryotic initiation factor eIF3 in promoting high REI competence of these two uORFs *in trans* (21). In particular, we found two separate regions within the a/TIF32-NTD, called Box 6 (amino acid residues 51–60) and 17 (residues 161–170), the mutations of which severely reduced REI permissiveness of both uORFs (22). Genetic epistatic experiments then revealed that RPE i and RPE iv of uORF1 and RPE v of uORF2 co-operate with both a/TIF32-NTD boxes in promoting efficient REI (22,30). The a/TIF32-NTD has a favorable location on the 40S subunit next to the mRNA exit channel (27,29,37,38), where it could theoretically come in direct contact with these RPEs that, upon termination on uORF1 or uORF2 stop codons, have already emerged from the exit pore and became solvent-exposed. However, more experiments are needed to reveal whether the co-operation between a/TIF32 Boxes and RPEs is based on direct or just a functional interaction. (RPEs ii and iii operate in the eIF3-independent manner and the molecular mechanism of their action is unknown). Collectively these findings led to a hypothesis that while the eIF3-bound 40S ribosome scans through the region upstream of uORF1 (or uORF2) and subsequently translates it—still bound by eIF3, the RPEs progressively fold into a specific secondary structure. Upon termination, eIF3 interacts with the corresponding RPEs to specifically stabilize only the small ribosomal subunit on the uORF1 (or uORF2) stop codon. Thanks to the incomplete ribosomal recycling, the post-termination 40S subunit can, upon acquisition of other essential eIFs, resume scanning for REI downstream. This process is a lot less efficient on uORFs 3 and 4 because they lack the RPEs and hence the eIF3-mediated 40S-stabilization effect is ineffective.

Here, we set out to thoroughly test this hypothesis. In particular, we wished to provide direct *in vivo* evidence that eIF3 remains bound to elongating ribosomes post-initiation, and in case of short uORFs that are REI-permissive, it interacts with their *cis*–acting features described above to render them highly REI competent. Toward this end, we developed a novel *in vivo* RNA–protein Ni^2+^-pull down (RaP-NiP) assay and showed that by pulling down eIF3 but not eIF2 a significantly higher amount of RNA segments encompassing uORF1 or uORF2 versus uORF3 or uORF4 are retrieved. Importantly, the efficiency of this enriched co-purification is strongly dependent on intact RPEs as well as the a/TIF32 Boxes 6 and 17. Mutating the AU-rich motif-containing 3΄ sequence immediately flanking the uORF1 stop codon likewise dramatically reduced the co-purification efficiency of the uORF1 RNA segment, and so did the gradual extension of uORF1. Based on these and other findings we propose and discuss the molecular model of translational control by reinitiation that relies on the short-lived retention of eIF3 on elongating ribosomes.

## MATERIALS AND METHODS

### Construction of plasmids and yeast strains

Construction of plasmids and yeast strains is described in the Supplementary Material.

### Yeast *in vivo* RNA–protein Ni^2+^-pull down (RaP-NiP) assay

To explore the nature of the previously reported functional interaction between the RPEs of uORF1 and uORF2 of *GCN4* and the NTD of the a/TIF32 subunit of eIF3 (22,30), we first had to create a yeast strain deleted for chromosomal *GCN4* (including its 5΄ UTR) and *TIF32* that we named YVM1 (its construction is described in the Supplementary information). The YVM1 strain was then transformed with the RaP-NiP plasmids (pMP29 to pMP57) and, in some cases, the wt *TIF32* gene carried on the covering plasmid was plasmid shuffled for its mutant alleles. The resulting transformants were cultured in the SD media (150 ml) supplemented with required amino acids to an OD_600_ ∼1, and the exponentially growing cells were split into two fractions: (i) one-third of the total volume was not formaldehyde cross-linked as it was used purely for isolation of total RNA by hot-phenol extraction as a control, and (ii) the remaining two-thirds of cells were subjected to yeast *in vivo* RNA–protein Ni^2+^-pull down assay (RaP-NiP).

The latter proportion of exponentially growing cells was first cross-linked with 1% formaldehyde at 4°C for 1.5 h and the cross-linking reaction was stopped using 125 mM glycine. The cells were collected in 200 ml polypropylene tubes by centrifugation (3,724 rcf for 6 min at 4°C), washed with 10 ml of ice-cold water, transferred into 15 ml falcon tubes and collected by centrifugation (1455 rcf for 3 min at 4°C). The cell pellet was weighed and resuspended in the 1:1 ratio (ml:g) in the Breaking Buffer (BB) composed of only nano-pure water supplemented with the protease inhibitor complete EDTA-free tablet (Roche), 1 mM PMSF, 30 mM Imidazole and 0.486 mM β-mercaptoethanol (interestingly, the best reproducibility of our RaP-NiP results was achieved when we used only nano-pure water lacking all classical salt constituents). The Whole Cells Extracts (WCEs) were prepared by breaking the cells manually using glass beads (∼1.3 volume) by vigorous vortexing (8 × 30 s with 1 min breaks on ice). The WCEs were collected by centrifugation at 3,274 rcf for 5 min at 4°C, transferred into fresh pre-cooled 1.5 ml Eppendorf tubes and cleared by two consecutive rounds of centrifugation at 16,100 rcf at 4°C for 2 and 10 min, respectively. The total protein content was estimated by the Bradford assay.

To prepare reactions for the RNase H digestion of the uORF constructs, 750 μg of total protein in 300 μl of BB was incubated with 15 μl of 100 μM sequence-specific custom made oligos (MP7 and MP79) at 37°C for 10 min. (before adding the oligos we always set aside 5 % (37.5 μg) of total protein as ‘Input’ for western blot analysis of our routine check-ups of the Ni^2+^-pull down efficiency of eIF3 subunits). The RNase H pre-reactions were then left at the room temperature for 10 min, after which 10 U of RNase H (NE Biolabs) was added for 20 min at 37°C. Careful optimization of the RNase H reaction conditions using the uORF1-only construct revealed that 15 μl of 100 μM oligos and 10 U of RNase H works the best in achieving the efficient digestion of the uORF1 specific transcript in WCEs (750 μg of total protein) when incubated at 37°C for 20 min.

For the Ni^2+^-pull down assay, we first took 15 μl of the 50% Ni-sepharose slurry (GE Health Care) in a fresh Eppendorf tube and washed the beads in 1 ml of BB three times (washes were separated by centrifugations at 500 rcf for 2 min at 4°C). Subsequently, the beads were incubated with the whole volume of RNase H-digested reactions supplemented with 3.3 U of SUPERase In™ RNAse Inhibitor (Ambion by Life technologies) overnight at 4°C by gentle shaking. The next day we first set aside 5% of the flow through (FT) for western blot analysis of our routine check-ups of the Ni^2+^-pull down efficiency, and then we washed the beads three times in 1 ml BB still supplemented with the same concentration of the RNAse inhibitor as above); washed beads were always collected by centrifugations at 500 rcf for 2 min at 4°C. The eIF3-co-purifying complexes were eluted in 50 μl of Elution Buffer (EB – 20 mM Tris–Cl, pH 7.5, 100 mM KCl, 5 mM MgCl_2_, 10% glycerol supplemented with the protease inhibitor complete tablet (1 tablet for 12.5 ml of buffer), 1 mM PMSF, 250 mM Imidazole, 0.486 mM β-mercaptoethanol and 2 U of SUPERase In™ RNAse inhibitor for 30 min at 4°C by gentle shaking. The resulting eluates were collected by centrifugation at 500 rcf for 2 min (at this step we always preserved 10 μl of eluate as ‘Elute’ for western blot analysis of our routine check-ups of the Ni^2+^-pull down efficiency, as described in (39)) and subsequently all proteins in the samples were digested with 0.8 U of Proteinase K (NE Biolabs) at 37°C for 30 min.

In the case of the eIF2γ Ni^2+^-pull down assay, the YMP34 strain was transformed with the selected RaP-NiP constructs along with the *GCD11-His* allele-carrying vector (pMP65) (40) and the resulting transformants were cultured in the SD media and subjected to the RaP-NiP as described above.

All captured RNAs, as well as total RNAs from WCEs derived from the formaldehyde untreated control cells, were purified by hot phenol extraction as follows. The WCEs from the formaldehyde untreated control cells were prepared in the same way as described for cross-linked cells except that the cells were broken using the FastPrep-24 cell homogenizer from MP Biomedicals using the following program: MP: 2 × 20, at speed 5 M/S for 40” at 4°C. The Proteinase K digested Eluates as well as WCEs from the un-cross-linked cells were adjusted to 400 μl with the TES solution prepared in DEPC-treated nano-pure water to a final concentration of 10 mM Tris–Cl, pH 7.5, 10 mM EDTA, 0.5 % SDS and incubated with acid phenol in 1:1 ratio at 65°C for 10 min with vigorous vortexing for 10 s every 5 min. The samples were left on ice for 5 min and subsequently centrifuged at 16,000 rcf for 5 min at 4°C. The aqueous phase was transferred into a clean RNase-free Eppendorf tube and once again cleared with acid phenol by vigorous vortexing for 10 s and centrifuged as before. The aqueous phase was again transferred into a clean RNase-free Eppendorf tube and the trace amounts of phenol were removed by mixing the aqueous phase with Chloroform (1:1) and vigorously vortexing for 10 s. The aqueous phase was collected as mentioned above and RNAs were precipitated by mixing the aqueous phase with 40 μl of 3M sodium acetate (pH 5.3) and 1 ml of ice-cold 100% ethanol and incubating the reactions at –80°C overnight. The RNA precipitates were the next day pelleted by centrifugation (16,000 rcf for 30 min at 4°C); the pellets were washed with ice-cold 70% ethanol and air dried.

The resulting RNA pellets were resuspended in DEPC-treated water along with the RNAse free DNAse buffer and the resulting samples (29 μl) were incubated with 1 μl (2 U) of DNAse I (2000 U/ml; NE Biolabs) at 37°C for 1 h to digest all contaminating DNAs. Subsequently, 1 μg of total RNA and 8 μl of captured RNA were used for the cDNA synthesis using the High Capacity cDNA Reverse Transcription kit (Applied Biosystems). All samples were analyzed for the amounts of co-purifying uORF RNA transcripts using qPCR as follows. RNA amounts were quantified by NanoDrop 2000/2000c (Thermo Scientific).

qPCR was carried out according to manufacturer's instructions (Solis BioDyne). qPCR reactions were prepared by mixing 5× HOT FIREPol^®^ EvaGreen^®^qPCR Mix Plus (no ROX) (Solis BioDyne) with 0.8 μM primers and 3 μl of 10-times diluted cDNAs and run using the following program: 95°C for 15΄ followed by 45 cycles of 95°C for 15”, 62°C for 20” and 72°C for 30”. Melting curves were analyzed between 65 and 95°C. Recovered amounts of specific RNA segments were normalized to both a recovered reference *ACT1* mRNA, as well as to the total RNA levels, to avoid any potential errors such as unexpected changes in expression levels of uORF mRNA constructs in the cells and/or inconsistencies in the Ni^2+^-pull downs. Please note that the MIQE specification guidelines as defined by the journal were closely followed.

### Other techniques

The β-galactosidase assay was carried out as described before (41).

## RESULTS

### Development of the yeast *in vivo* RNA–protein Ni^2+^-pull down (RaP-NiP) assay

**Figure 1.**
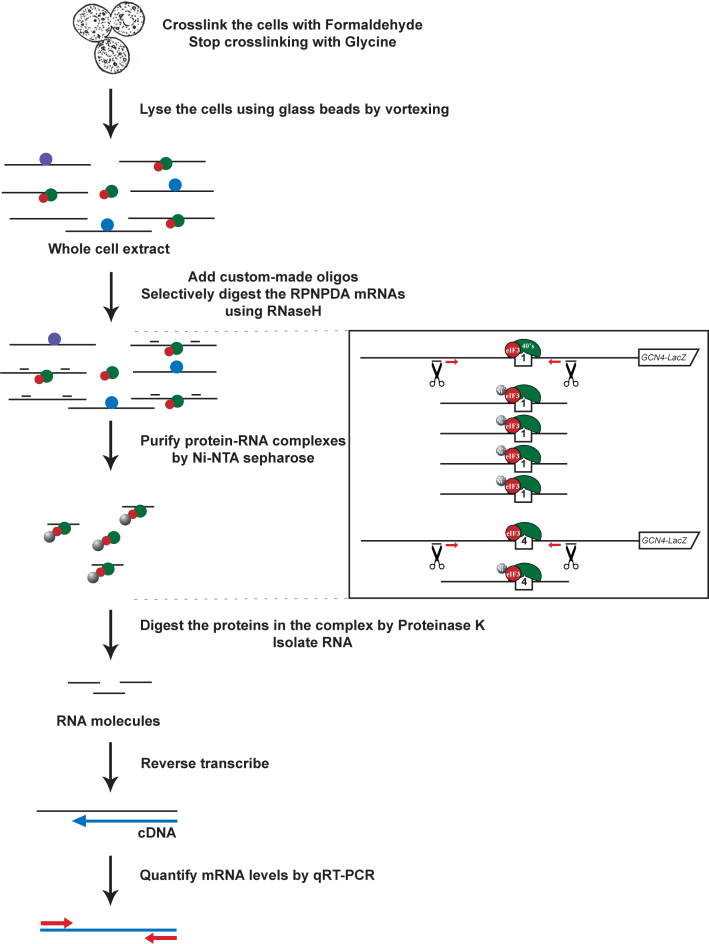
Schematic representation of yeast *in vivo* RNA–protein Ni^2+^-pull down (RaP-NiP) assay using formaldehyde crosslinking. The basic scheme of the RaP-NiP is described in the form of a flowchart. Green and red balls represent 40S ribosomes and eIF3 complexes, respectively, grey balls stand for the Ni^2+^ beads, and purple and blue balls depict some non-specific RNA binding proteins. Exponentially growing yeast cells were crosslinked with 1% formaldehyde. Crosslinking was stopped by adding glycine and the fixed cells were lysed using glass beads by rigorous vortexing. Pre-cleared whole cell extract (WCE) containing RaP-NiP mRNAs in protein-RNA complexes were selectively digested with RNase H using sequence specific custom-made oligos. The resulting specific mRNA segments were purified with the help of the His-tagged a/TIF32 subunit of yeast eIF3 or its mutant variants using the Ni-NTA sepharose beads. Thus isolated protein-RNA complexes were subsequently treated with Proteinase K, and the captured RNAs were further purified by hot phenol extraction, reverse transcribed and their amounts were then quantified by qRT-PCR. The schematic boxed on the right-hand side illustrates typical amounts of RNAse H digested RNA segments of REI-permissive uORF1 and REI-non-permissive uORF4 from the *GCN4* mRNA leader co-purifying with eIF3, the typical ratio of which is ∼4:1.

In order to unambiguously demonstrate that eIF3 remains bound to elongating ribosomes post-initiation and that the RPEs of uORF1 and uORF2 do interact with the NTD of a/TIF32 *in vivo* to promote REI, as predicted from our previous genetic experiments (21,22), we developed a novel method combining approaches from several existing assays such as the human RIP assay, the CLIP assay, the yeast 43–48S PIC formaldehyde cross-linking assay and the Ni^2+^ affinity chromatography assay (39,42–44), which we call yeast *in vivo*RNA–Protein Ni^2+^-Pull Down (RaP-NiP) assay (Figure 1). In short, specific constructs described below were individually introduced into yeast cells that were (i) deleted for chromosomal *GCN4* and its 5΄ flanking sequences and (ii) expressing a His-tagged *TIF32* subunit of eIF3 as the sole allele of this gene. Exponentially growing transformants were cross-linked with low concentration formaldehyde (1%), the whole cell extracts (WCE) were prepared and pre-incubated with a set of two sequence-specific custom-made oligonucleotides for the subsequent RNase H cleavage, shown to be highly specific (Supplementary Figure S3A and B). Thus, digested samples were incubated with Ni^2+^-sepharose beads to pull down eIF3 and any co-purifying proteins and RNAs (Supplementary Figure S3C). The complexes were then incubated with Proteinase K to digest all proteins, and co-purifying RNA was isolated by hot phenol extraction and subsequently treated with DNase I to remove any DNA contamination (Supplementary Figure S3D). cDNAs were synthesized by RT-PCR and the relative co-purification yields were determined by qPCR. All samples were normalized to the amounts of corresponding input (WCE) mRNA levels as well as to the *ACT1* house-keeping gene, the mRNA of which was also recovered in trace amounts by this procedure.

**Figure 2.**
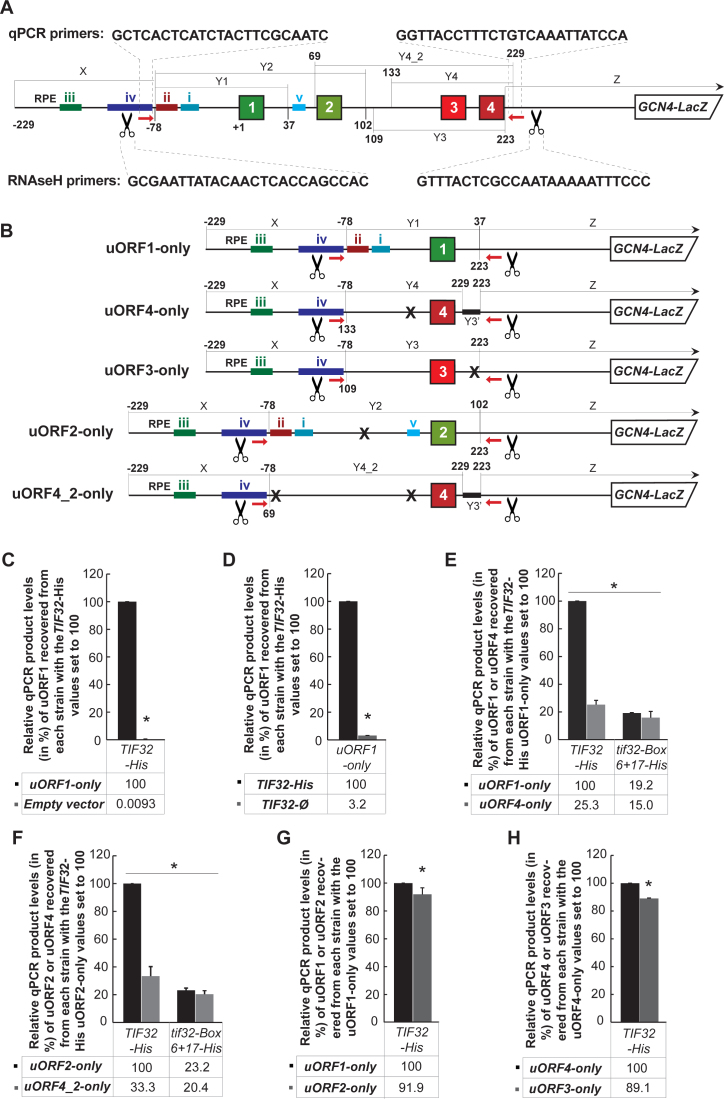
eIF3 stabilizes the post-termination 40S complexes on stop codons of REI-permissive uORF1 and uORF2 from the *GCN4* mRNA leader. (**A**) A schematic showing the wild type mRNA leader of the *GCN4-lacZ* fusion with colored bars indicating positions of individual RPEs of uORF1, as well as of uORF2 (color coding of all four uORFs reflects their REI-permissiveness (green) or -non-permissiveness (red)—for details see Supplementary Figures S1 and S2). The mRNA leader was divided into several segments (X, Y_*n*_ and Z), where X and Z are present in all constructs shown in panel B and contain the RNase H cutting sites (indicated by scissors) and qRT-PCR primer binding sites (indicated by red arrows) at their 5΄ and 3΄ ends, respectively. The segment X is 151 bp in length (from position –229 to –79 relative to the uORF1 AUG start codon) and the segment Z encompasses the entire downstream sequence immediately following the uORF4 stop codon (i.e. from position +223 relative to the uORF1 AUG start codon downstream). The coordinates of all Y segments, by which the constructs in panel B differ and that are in each of them placed between the X and Z segments, are given at the top or bottom of the schematic. (**B**) Schematics showing individual uORF1–4 RaP-NiP constructs with corresponding Yn inserts of the same length for uORF1-only, 3-only and 4-only constructs (top three), and for the uORF2-only and uORF4_2-only constructs (bottom two). Black bars labeled as Y3’ represent composite 13+5 nt taken from the uORF3 3΄ UTR that were placed immediately behind the first 7 nt of the uORF4 3΄ UTR (i.e. behind the Y4 segment that ends exactly at the seventh nt of the uORF4 3΄ UTR) to keep the length of the uORF4-specific Y segment the same as that of uORF1. (We could not take the entire 3΄ UTR of uORF4 because in our set-up it is an integral part of the Z segment where the downstream qPCR primer base-pairs). (**C** and **D**) The RNase H-cleaved uORF1 segment specifically co-purifies with the His-tagged a/TIF32 subunit of eIF3 using the *in vivo* RaP-NiP. (C) The YMP1 (*gcn4Δ TIF32-His*) strain was introduced either with the uORF1-only RaP-NiP construct shown in panel B or an empty vector and the resulting transformants were pre-cultured in minimal media overnight, diluted to OD_600_ ∼ 0.1 and further cultivated to OD_600_ ∼1. The exponentially growing cells were then subjected to RaP-NiP as described in Materials and Methods and outlined in Figure 1. Relative qPCR product levels (in %) of the Y1 segment of uORF1 recovered from each strain with standard deviations obtained from at least three independent experiments from three independent transformants (i.e. biological replicates) normalized to reference *ACT1* mRNA as well as to total RNA levels are given with the values of uORF1-only set to 100 (asterisks indicate that *P* < 0.05). (D) The YMP1 (*gcn4Δ TIF32-His*) and YMP6 (*gcn4Δ TIF32*) strains were introduced with the uORF1-only RaP-NiP construct shown in panel B and treated as described in panel C. Relative qPCR product levels (in %) of the Y1 segment of uORF1 recovered from each strain were processed as described in panel C with the values of *TIF32-His* set to 100 (asterisks indicate that *P* < 0.05). (**E**) eIF3 stabilizes the post-termination 40S complexes on the stop codon of REI-permissive uORF1. The YMP1 (*gcn4Δ TIF32-His*) and YMP28 (*gcn4Δ tif32-Box-6+17-His*) strains were introduced with the uORF1-only or uORF4-only RaP-NiP constructs shown in panel B and treated as described in panel C. Relative qPCR product levels (in %) of the corresponding Y1 or Y4 segments of uORF1 or uORF4 recovered from each strain were processed as described in panel C with the values of *TIF32-His* uORF1-only set to 100 (asterisks indicate that *P* < 0.05). (**F**) eIF3 stabilizes the post-termination 40S complexes on the stop codon of REI-permissive uORF2. The YMP1 and YMP28 strains described in panel E were introduced with the uORF2-only or uORF4_2-only RaP-NiP constructs shown in panel B and treated as described in panel C. Relative qPCR product levels (in %) of the corresponding Y2 or Y4_2 segments of uORF2 or uORF4 recovered from each strain were processed as described in panel C with the values of *TIF32-His* uORF2-only set to 100 (asterisks indicate that *P* < 0.05). (**G**) The eIF3 stabilization effect on post-termination 40S complexes is similar for both REI-permissive uORFs. The YMP1 strain described in panel C was introduced with the uORF1-only or uORF2-only RaP-NiP constructs shown in panel B and treated as described in panel C. Relative qPCR product levels (in %) of the corresponding Y1 or Y2 segments of uORF1 or uORF2 recovered from each strain were processed as described in panel C with the values of uORF1-only set to 100 (asterisks indicate that *P* < 0.05). (**H**) The eIF3 stabilization effect on post-termination 40S complexes is similarly weak for both REI-non-permissive uORFs. The YMP1 strain described in panel C was introduced with the uORF4-only or uORF3-only RaP-NiP constructs shown in panel B and treated as described in panel C. Relative qPCR product levels (in %) of the corresponding Y4 or Y3 segments of uORF4 or uORF3 recovered from each strain were processed as described in panel C with the values of uORF4-only set to 100 (asterisks indicate that *P* < 0.05).

Special attention was given to the design of the RaP-NiP constructs. To compare the eIF3 occupancy on terminating 80S ribosomes at REI-permissive versus REI-non-permissive uORFs, we prepared constructs individually carrying each of the four *GCN4* uORFs flanked by their genuine 5΄ and 3΄ sequences in a single copy vector (Figure 2A and B). The idea was that when the eIF3-bound elongating 80S ribosome terminates at the stop codon and undergoes recycling, the RPEs preceding only uORF1 or uORF2 will interact with the a/TIF32-NTD in order to stabilize the 40S subunit specifically on stop codons of these REI-permissive uORFs but not on stop codons of REI-non-permissive uORFs 3 and 4. Cutting out the short RNA segments encompassing individual uORFs and their flanking sequences by RNase H and pulling them down by eIF3 should thus retrieve significantly higher amounts of uORF1 or uORF2 RNA segments, respectively, compared to uORF3 or uORF4 segments. Cross-linking with a low concentration of formaldehyde should preserve these most probably only shortly lived eIF3-40S-post-termination complexes with minimal negative impact on the RNase H digestion. (The formaldehyde treatment is known to produce the Schiff's base adducts between formaldehyde and the amino groups of the nucleotides in single-stranded regions of the mRNA thus preventing the proper base-pairing of oligonucleotides to the mRNA, which is indeed necessary for the efficient RNase H cleavage.) That is why we used as low of a concentration of formaldehyde and as short of an incubation time as possible, simply assuming that a fraction of uORF-carrying mRNAs will stochastically have the sequences flanking the eIF3 binding site available for base pairing with oligonucleotides for the RNase H cleavage. Indeed, we observed that the cutting efficiency was only modestly decreased in 1% formaldehyde cross-linked *versus* uncrosslinked WCEs (Supplementary Figure S3A; compare 15.57% versus 9.6% between panels i and ii for the uORF1-only construct; or Supplementary Figure S3B; compare 26.7% versus 4.3% between panels i and ii for the uORF4-only construct), and that even a modest increase in the formaldehyde concentration (from 1% to 2%) significantly reduced the efficiency of our assay (data not shown). In addition, optimizing the reaction conditions for the RNAse H digestion using the uORF1-only construct revealed that the best results are obtained when the WCE (750 μg of total protein estimated by Bradford) are supplemented with 1.5 nmol RNase H cutting oligonucleotides of 25 nt in length together with 10 U of the RNAse H enzyme, and the reactions are carried out at 37°C for 20 min.

In order to minimize the RNase H cutting and qPCR amplification errors among mRNAs carrying different uORFs, we took the uORF1-only construct shown in Figure 2B as a template, designed the specific 5΄ and 3΄ RNase H cutting and qPCR amplification primers, and subsequently replaced the uORF1 segment (encompassing the 5΄ and 3΄ REI-promoting elements shown in Supplementary Figure S2) bordered by both sets of primers (Y1 in Figure 2A) with the corresponding segments of uORF3 and uORF4 (Y3 and Y4 in Figure 2A; please note that the uORF4-only construct has the AUG of uORF3 mutated out; for more details on various segments please see the corresponding Fig. legend). This way the RNase H cutting, as well as qPCR primers, were the same for all three constructs and, intuitively, so was the length of the qPCR amplicon (Figure 2B). The identical length of the RNase H digested fragment for all these uORFs is critical because segments with varying lengths could introduce a ribosome occupancy error. In other words, we expected that eIF3-bound 40S ribosomes scanning through the 5΄ UTRs will be also pulled down in our procedure, with the relatively same efficiency among all RaP-NiP constructs (see below). Therefore generating RNA segments of uneven size could result in varying ribosome occupancy that would artificially influence the amounts of the RNase H-cleaved RNAs co-purifying with the His-tagged eIF3, which would not directly reflect the true differences among the uORF constructs. Please also note that due to this arrangement, the uORF1-specific RPEs iii. and iv. were preserved in the uORF3-only and uORF4-only constructs (Figure 2B). As expected, this fact had no effect on the efficiency of co-purification of their RNA segments because RPE iii. contributes only modestly to the overall REI-promoting activity of uORF1, and RPE iv. is non-functional in the absence of RPE i. (22). For the uORF2-only construct we wished to leave its entire 5΄ leader sequence intact (with the exception of AUG of uORF1 that had to be mutated out), and thus the length of the uORF2-specific amplicon is longer by 65 nt compared to the uORF1-specific amplicon; importantly, the RNase H, as well as the qPCR primer sites, remained unchanged (Figure 2B). As a control, we also prepared the second uORF4 construct (uORF4_2-only) with the arrangement analogous to that of uORF2 (Figure 2B; please note that this uORF4 construct has AUGs of both uORF2 and uORF3 mutated out). Importantly, functionality—mainly with respect to the permissiveness for REI - of our RaP-NiP constructs with the beginning of the *GCN4* gene fused in-frame with bacterial *LacZ* was verified in β-galactosidase assays (Supplementary Figure S4A–D).

To demonstrate the high specificity and reproducibility of our RaP-NiP method, we examined the efficiency of co-purification of the RNase H-cleaved uORF1 segment from the *gcn4Δ TIF32-His* cells expressing the uORF1-specific RaP-NiP construct compared to cells expressing only an empty vector. As shown in Figure 2C, cells expressing an empty vector produced only a noise signal. Similarly, a background 3% of the specific RNase H-cleaved uORF1 segment was recovered from *gcn4Δ* cells expressing untagged *TIF32* compared to its His-tagged allele (Figure 2D). All presented experiments were repeated several times with at least three biological replicates.

### eIF3 stabilizes the post-termination 40S complexes on stop codons of REI-permissive uORF1 and uORF2 from the *GCN4* mRNA leader

Having developed the RaP-NiP assay, we first wished to compare the occupancy of the eIF3-bound post-termination 40S ribosomes individually at all four uORFs occurring in the *GCN4* mRNA leader. As mentioned above, since eIF3 co-operates with uORF1- and uORF2-specific RPEs to promote efficient REI (21,22,30), we rationalized that co-purification of RNA segments containing these two REI-permissive uORFs should be higher compared to co-purification of RNA segments bearing REI-non-permissive uORFs 3 and 4. Consistent with our rationale, we detected ∼4-fold higher amounts of the uORF1-specific RNA segment co-purifying with His-tagged a/TIF32 compared to the uORF4-specific segment (Figure 2E). Similarly, ∼3-fold higher amounts of the uORF2-specific RNA segment were retrieved compared to uORF4 (Figure 2F). Importantly, introducing the *Box 6+17* mutation, known to disrupt the a/TIF32 interaction with RPEs of uORF1 and uORF2 (22,30), into the *TIF32-His* allele markedly diminished the difference in the efficiency of RNA co-purification between uORF1 or uORF2 and uORF4 (Figure 2E and F). These data strongly suggest that the active contact between the intact a/TIF32 Box 6 and 17 amino acid residues and the RPEs is required to stabilize post-termination 40S ribosomes on the *GCN4* mRNA.

A side by side comparison of the RNA co-purification efficiency between REI-permissive uORF1 and uORF2 (Figure 2G) and REI-non-permissive uORF3 and uORF4 (Figure 2H) showed only a modest differences. This data is perfectly consistent with the roles of uORFs 1 and 2 versus uORFs 3 and 4 in the *GCN4* translational control mechanism.

**Figure 3.**
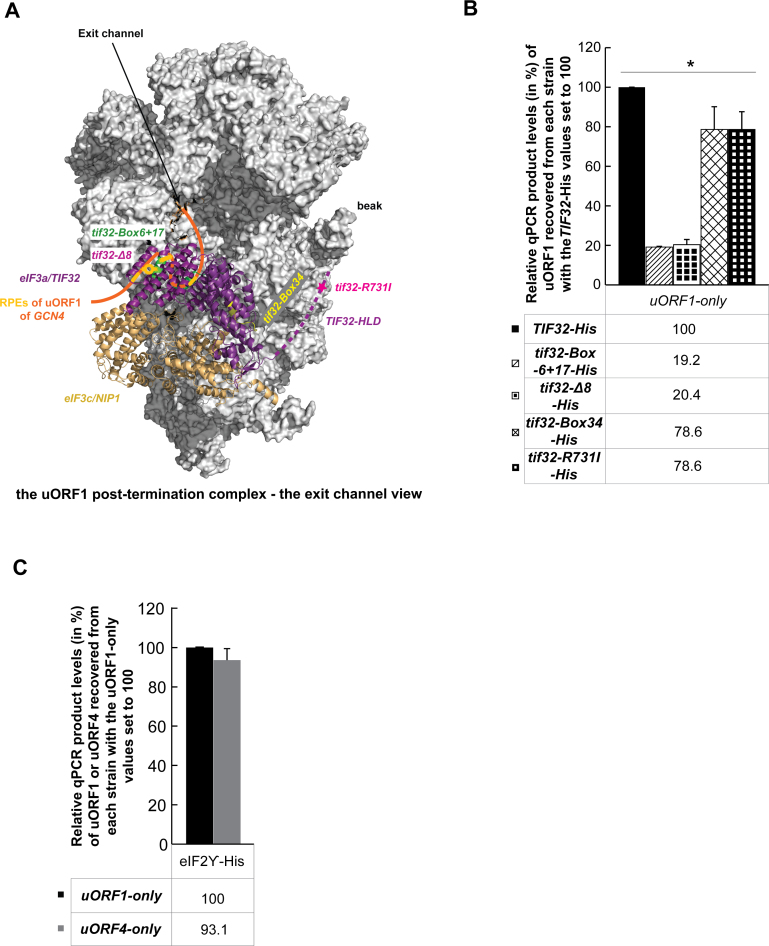
The eIF3 stabilization effect on post-termination 40S complexes is not significantly impaired in a/TIF32 mutants with no REI phenotype, and the RaP-NiP carried out with the His-tagged eIF2γ showed no difference between uORF1 and uORF4. (**A**) Graphical illustration of the proposed arrangement of the post-termination complex on uORF1 with its RPEs interacting with Box 6 and Box 17 segments of the N-terminal domain of a/TIF32 to promote resumption of scanning for REI on *GCN4*. The exit channel view of the py48S-closed complex (adopted from (29)) shows only two incomplete eIF3 subunits for simplicity: eIF3c/NIP1 in wheat and eIF3a/TIF32 in purple with its extreme NTD in light purple (entirely missing in *tif32-Δ8*) and its C-terminal HCR1-like domain (HLD) represented by a dotted line (its structure is unknown and thus its placement in the py48S complex was only predicted). The location of each *tif32* mutation used in panel B is marked; tif32-*Box 6*+*17* in green, *tif32-Box 34* in yellow and *tif32-R731I* with a star in pink. The 5΄-UTR of uORF1 is shown in orange with its RPEs depicted in yellow-orange. (**B**) The YMP1 and YMP28 strains described in Figure 2E as well as YMP77 (*gcn4Δ tif32-Δ8-His*), YMP10 (*gcn4Δ tif32-Box 34-His*) and YMP9 (*gcn4Δ tif32-R731I-His*) strains were introduced with the uORF1-only RaP-NiP construct shown in Figure 2B and treated as described in Figure 2C. Relative qPCR product levels (in %) of the Y1 segment of uORF1 recovered from each strain were processed as described in Figure 2C with the values of *TIF32-His* set to 100 (asterisks indicate that *P* < 0.05). (**C**) eIF2 shows no preference in association with RNA segments encompassing either uORF1 or uORF4. The YMP34 (*gcn4Δ TIF32)* strain was introduced with the uORF1-only or uORF4-only RaP-NiP constructs shown in Figure 2B along with pMP65 carrying all three subunits of eIF2 with *GCD11* allele bearing a His tag and treated as described in Figure 2C. Relative qPCR product levels (in %) of the corresponding Y1 or Y4 segments of uORF1 or uORF4 recovered from each strain were processed as described in Figure 2C with the values of *GDC11-His* uORF1-only set to 100.

To demonstrate that the observed defect of the aforementioned a/TIF32 mutant in the stabilization of post-termination 40S ribosomes at uORFs 1 and 2 is specific, we examined two additional a/TIF32 mutants with mutations lying outside of the a/TIF32 REI-promoting region, as illustrated in Figure 3A. In particular, we measured the efficiency of the RNA segment co-purification of the uORF1-only construct in *tif32-R731I*, previously shown to affect mRNA recruitment, scanning and AUG recognition of the 48S PICs during primary initiation event (45–47), and in *tif32-Box 34* (residues 331–341) with a yet to be described initiation defect that most probably involves a modest impairment of mRNA recruitment (48). Importantly, neither of these mutations impair the efficiency of resumption of scanning for REI downstream ((46) and data not shown). As can be seen in Figure 3B, both of these mutations had a lot milder effect on the efficiency of the uORF1-specific segment co-purification when compared to the REI-specific *Box 6+17* mutant (compare ∼20% to ∼80% reduction). In contrast, another REI-deficient mutation in the a/TIF32-NTD that removes its first 200 amino acid residues, known as the *tif32-Δ8* mutation (21,37), displayed as a dramatic defect as the *Box 6+17* mutation (Figure 3B). The minor reduction (∼20%) seen with the former two *tif32* mutations could be attributed to their defects in general translation initiation, for example in the mRNA recruitment step.

To rule out that the 40S ribosomes that are scanning towards the uORFs from the 5΄ cap could be creating the differences observed among our constructs, as opposed to the post-termination 40S ribosomes that have already translated the uORFs, we performed the RaP-NiP assay with the uORF1- and uORF4-specific constructs in *gcn4Δ* cells expressing all three eIF2 subunits on top of the endogenous genes, with the *GCD11* (γ) subunit carrying a His tag. The eIF2 factor is—upon AUG recognition followed by irreversible GTP hydrolysis - known to dissociate from the PICs to allow subunit joining, because it sits in the P-site of the small subunit (29) and so its presence post-AUG selection would sterically hinder the incoming 60S subunit. This contrasts with eIF3 occurring on the solvent-exposed side of the 40S subunit, as mentioned above. With respect to REI, eIF2 is clearly dispensable for the resumption of movement of post-termination ribosomes along mRNA Hence we rationalized that if we pull down the His-tagged eIF2-complex instead of the His-tagged eIF3-complex, the difference in the efficiency of the RNA segments co-purification between the strains carrying the uORF1- or uORF4-only constructs should be diminished, because only the primarily scanning 40S ribosomes will be targeted. This is exactly what we observed (Figure 3C). In addition, the same was true when we tested the selected uORF1 or uORF2 Rap-NiP mutants (Supplementary Figure S5). Taken together, our findings thus not only provide clear biochemical evidence directly implicating eIF3 in stabilizing the post-termination 40S complexes in order to stimulate efficient resumption of scanning from stop codons of the REI-permissive uORFs, but also strongly support the idea that eIF3 does remain bound to post-initiation 80S ribosomes for at least a few elongation cycles (see below).

### Evidence that the uORF1 RPEs co-operate with each other and with eIF3 to stabilize the 40S ribosomes terminating *in vivo* at the uORF1 stop codon

**Figure 4.**
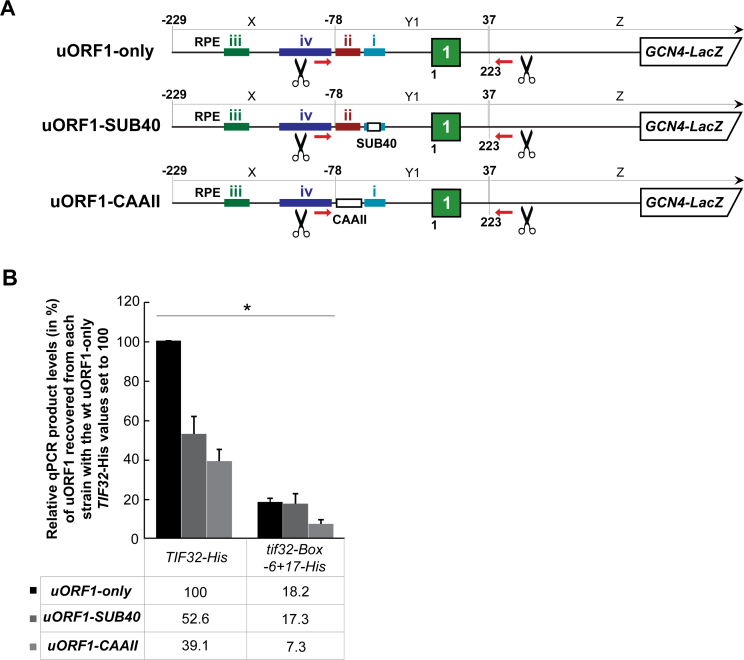
Evidence that the uORF1 RPEs co-operate with each other and with eIF3 to stabilize the 40S ribosomes terminating *in vivo* at the uORF1 stop codon. (**A**) Schematics showing uORF1-only RaP-NiP constructs with substitutions in RPE i. (uORF1-SUB40; substitution of nt -40 through -49 of RPE i with the complementary sequence) or in RPE ii (uORF1-CAAII; substitution of whole RPE ii. sequence [nt -55 through -76] with seven CAA repeats). (**B**) The YMP1 and YMP28 strains described in Figure 2E were introduced with the uORF1-only RaP-NiP construct or its derivatives shown in panel A and treated as described in Figure 2C. Relative qPCR product levels (in %) of the Y1 segment of uORF1 recovered from each strain were processed as described in Figure 2C with the values of the wt uORF1-only *TIF32-His* set to 100 (asterisks indicate that *P* < 0.05).

Having demonstrated that the mutated residues in Boxes 6 and 17 of a/TIF32 drastically reduced co-purification of the uORF1 RNA segment with eIF3, we next examined the effects of eliminating the uORF1-specific RPEs, some of which co-operate with these Boxes in promoting REI (22). In particular, we introduced previously identified mutations SUB40 and CAAII (22) into the eIF3-dependent RPE i. and eIF3-independent RPE ii., respectively, in our uORF1-specific RaP-NiP construct (Figure 4A). Both mutations significantly (by ∼50% and 60%, respectively) reduced the efficiency of the uORF1 RNA co-purification in *TIF32-His* wild type (wt) cells (Figure 4B), which perfectly correlates with our previous measurements of their partially impaired REI-promoting activity (22). Importantly, while the CAAII mutation further reduced (by ∼2.5-fold) the already compromised co-purification efficiency in *tif32-Box 6+17-His* cells, SUB40 showed epistatic effect (Figure 4B). These findings provide a strong *in vivo* support for our previous, genetic-based conclusions that RPE i. promotes REI by interacting with the a/TIF32-NTD, whereas RPE ii stimulates REI in the eIF3-independent manner that is yet to be elucidated. Nevertheless, the fact that the CAAII mutation of the otherwise structured RPE ii reduced the uORF1 RNA co-purification efficiency by eIF3 even though RPE ii operates in the eIF3-independent fashion indicates that its primary molecular role is likewise to stabilize post-termination 40S ribosomes on the mRNA. Hence it seems that all RPEs contribute by different means to the same goal.

### Evidence that the stabilization effect of eIF3 on 40S ribosomes terminating *in vivo* at the uORF2 stop codon is dependent on its eIF3-dependent RPE v

**Figure 5.**
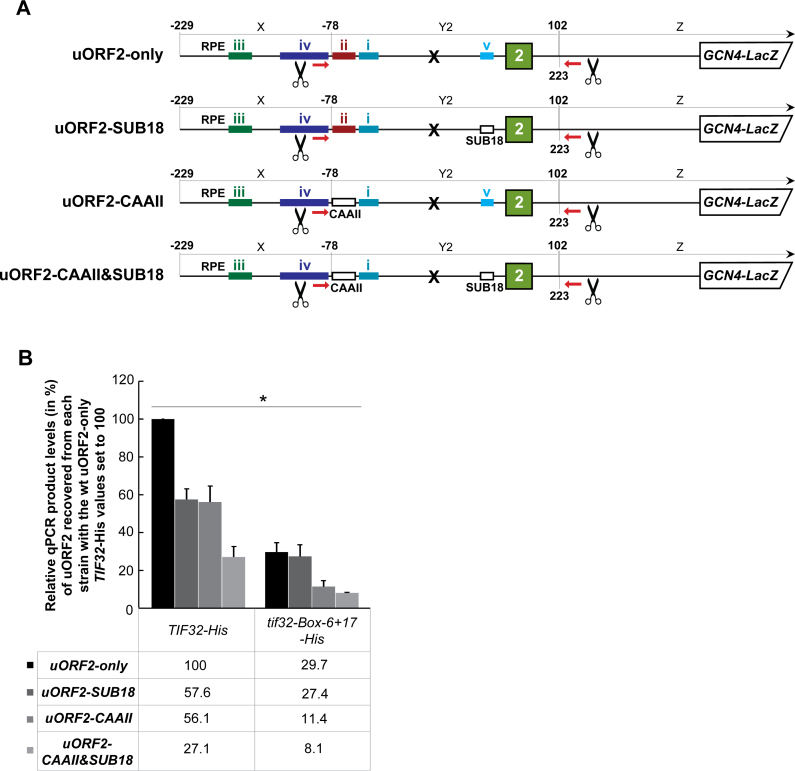
Evidence that the stabilization effect of eIF3 on 40S ribosomes terminating *in vivo* at the uORF2 stop codon is dependent on its eIF3-dependent RPE v. (**A**) Schematics showing uORF2-only RaP-NiP constructs with substitutions in RPE ii (uORF2-CAAII; described in Figure 4A) or in RPE v (uORF2-SUB18; substitution of nt +41 through +50 of RPE v with the complementary sequence) or of their combination (uORF2-SUB18&CAAII). (**B**) The YMP1 and YMP28 strains described in Figure 2E were introduced with the uORF2-only RaP-NiP construct or its derivatives shown in panel A and treated as described in Figure 2C. Relative qPCR product levels (in %) of the Y2 segment of uORF2 recovered from each strain were processed as described in Figure 2C with the values of the wt uORF2-only *TIF32-His* set to 100 (asterisks indicate that *P* < 0.05).

In analogy with the previous chapter, we also investigated the effects of eliminating the uORF2 RPEs; in particular RPE ii that both REI-permissive uORFs share and RPE v, which operates in the eIF3-dependent fashion (30). To do that, we introduced previously identified mutations SUB18 and CAAII (22,30) individually or in combination into the eIF3-dependent RPE v and eIF3-independent RPE ii., respectively, in our uORF2-specific RaP-NiP construct (Figure 5A). Both mutations significantly (by ∼40–45%) reduced the efficiency of the uORF2 RNA co-purification in *TIF32-His* wt cells when tested individually, their combination then displayed strong additive effect (Figure 5B). This is all in perfect agreement with our previous genetic measurements (30). Accordingly, whereas the SUB18 mutation showed genetic epistasis with the *Box 6+17* mutation, the CAAII mutation alone or in combination with SUB18 further reduced (by ∼3-fold) the already compromised co-purification efficiency in *tif32-Box 6+17-His* cells (Figure 5B). These data again clearly document that the RPE v of uORF2 promotes REI by interacting with the a/TIF32-NTD and further underscore that RPE ii additionally, contributes to the stabilization of post-termination 40S ribosomes on both REI-permissive *GCN4* uORFs in a manner that is completely independent of eIF3.

### The molecular action of the intact 3΄ AU-rich motif of uORF1 precedes and is indispensable for the following stabilizing function of the uORF1 RPEs

**Figure 6.**
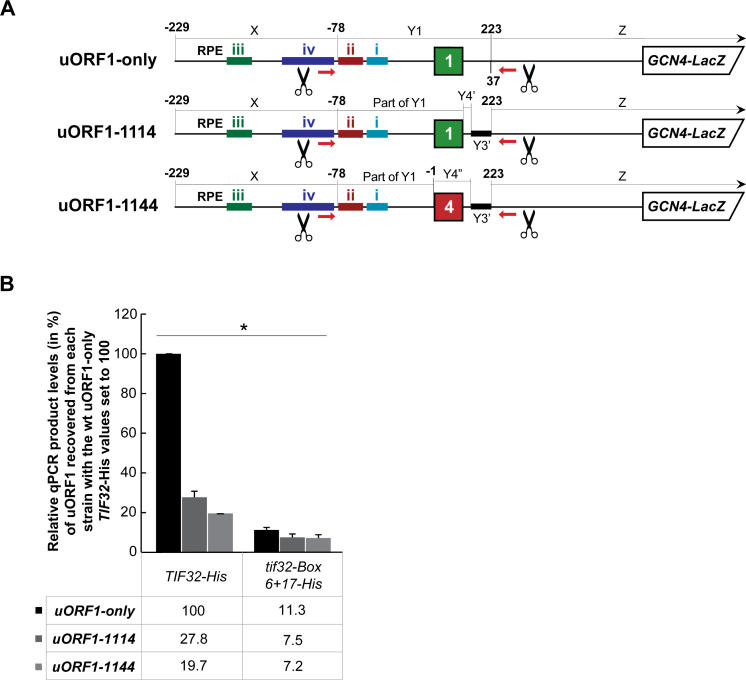
The intact 3΄ AU-rich motif of uORF1 is required for the REI-promoting action of its RPEs. (**A**) Schematics showing uORF1-only RaP-NiP constructs with substitutions of its 3΄ UTR (uORF1-1114; the first 25 nt immediately following the uORF1 stop codon were replaced with the corresponding sequence of the uORF4-only construct [Y4’+Y3’] shown in Figure 2B) or of its coding region together with its 3΄ UTR (uORF1-1144; the entire uORF1 coding region plus the first 25 nt immediately following the uORF1 stop codon were replaced with the corresponding sequences of the uORF4-only construct [Y4’’+Y3’] shown in Figure 2B). (**B**) The YMP1 and YMP28 strains described in Figure 2E were introduced with the uORF1-only RaP-NiP construct or its derivatives shown in panel A and treated as described in Figure 2C. Relative qPCR product levels (in %) of the Y1 segment of uORF1 recovered from each strain were processed as described in Figure 2C with the values of the wt uORF1-only *TIF32-His* set to 100 (asterisks indicate that *P* < 0.05).

The molecular mechanism by which the AU-rich *cis*-acting element occurring in the first 12 nt immediately following stop codons of uORFs 1, 2 and 3 promotes REI is unknown (36). What was shown is that it promotes REI independently of other REI-promoting *cis*-acting features, but only when situated at the defined distance from the *GCN4* AUG start codon - in principle corresponding to the position of uORF1. That is why only uORF1 can utilize it (36). In addition, it was shown that it also operates in an eIF3-independent manner and, moreover, that its REI-promoting action precedes and actually serves as a prerequisite for the subsequent action of the RPEs (22). We became curious to examine what happens with the stability of the post-termination 40S–eIF3 complex if we mutate the AU-rich element. There are two possibilities: 1) in the absence of the AU-rich motif the RPEs in co-operation with the a/TIF32-NTD will no longer be able to efficiently stabilize the post-termination 40S subunit on the uORF1 stop codon; i.e. we should see a reduction in the recovery; 2) the stabilization effect will persist but the lack of the molecular action that the AU-rich motif normally ensures will prevent the post-termination 40S subunit from resumption of scanning; i.e. little to no reduction should be seen. To distinguish these possibilities, we replaced the 3΄ sequence of uORF1 with the corresponding hybrid sequence of uORF4 and uORF3 in the uORF1-specific RaP-NiP construct to preserve the same length (Figure 6A; 1114) and, in a separate construct, we additionally replaced the uORF1 coding region with that of uORF4 (Figure 6A; 1144). The coding region of uORF1 was also demonstrated to contribute to the high propensity of uORF1 for REI (36). Whereas the 1114 mutation reduced the efficiency of the uORF1 RNA co-purification by ∼3.6-fold, the combined 1144 mutation further exacerbated the decrease in co-purification efficiency down to ∼5-fold (Figure 6B). Expressing both mutations in the *tif32-Box 6+17-His* mutant then produced a similar ∼35% drop compared to uORF1-only expressing mutant cells (Figure 6B). Therefore these results further support our earlier idea that the still unknown molecular action of the 3΄ AU-rich motif precedes and is indispensable for the following stabilizing function of RPEs and the a/TIF32-NTD (22). In addition, they also nicely support our earlier conclusion that all three tested *cis-*acting uORF1 features, namely the RPEs, its coding sequence and the AU-rich motif, make at least to a certain degree, independent contributions to the overall REI potential of uORF1 (36).

### eIF3 travels with elongating 80S ribosomes on short uORFs to participate in termination and post-termination events

Even though all of the so far presented results, as well as our earlier genetic data, strongly support the idea that eIF3 remains bound to the 40S subunit after subunit joining and travels with 80S couples for a few elongation cycles during which it gradually drops off, we next asked whether the newly developed RaP-NiP assay can provide ultimate biochemical evidence for this case. Our previous genetic experiments revealed that lengthening of the 3-codons long uORF1 by up to 10 codons dramatically decreased the REI efficiency of extended uORF1. We hypothesized that two relatively independent effects contributed to this phenomenon on terminating ribosomes: (i) displacement of the eIF3-dependent RPEs out of reach of the mRNA exit channel-based a/TIF32-NTD (Figure 3A)—this effect dominated with shorter extensions (by two and three codons) and (ii) gradual dissociation of eIF3 from elongating ribosomes—this effect prevailed with longer extensions (by 5 and more codons) (21). In contrast, the readout of the lengthening of the 3-codons long uORF4—lacking any REI-promoting features—should be by definition influenced only by the latter effect (it is important to note here that eIF3 ensures very basic REI levels of any short uORF (2)).

**Figure 7.**
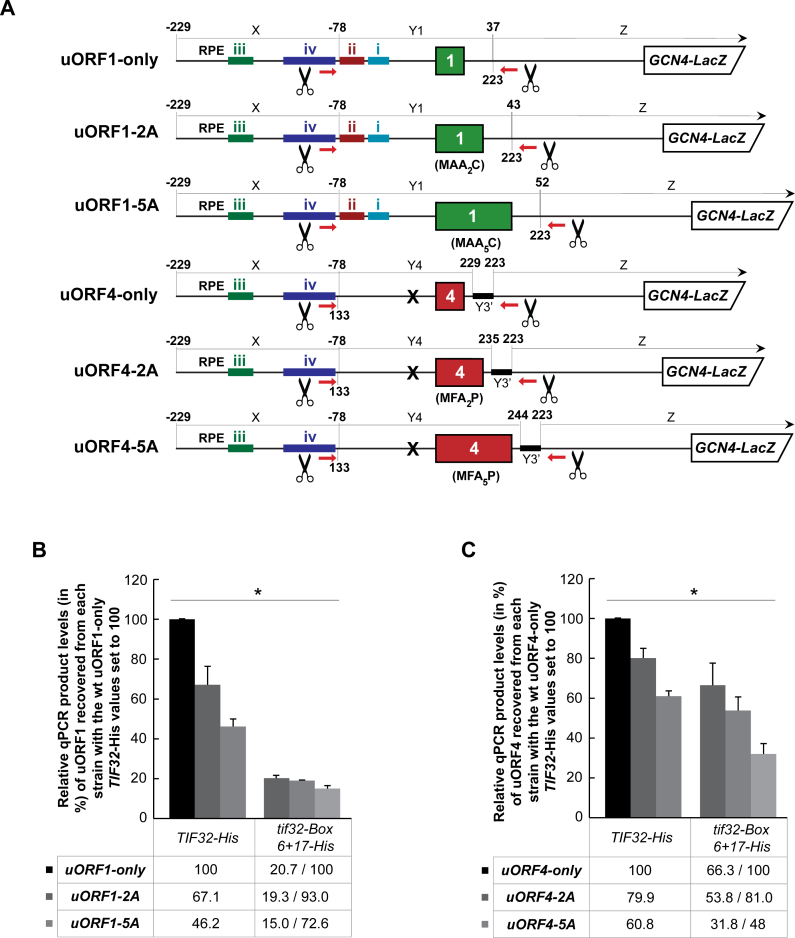
eIF3 travels with elongating 80S ribosomes on short uORFs to participate in termination and post-termination events. (**A**) Schematics showing individual uORF1 or uORF4-only RaP-NiP constructs with their CDS extended by two or five codons. The 3΄ end coordinates of the Y1 or Y4 fragments reflect the corresponding extensions of both uORFs. (**B**) The YMP1 and YMP28 strains described in Figure 2E were introduced with the uORF1-specific RaP-NiP constructs shown in panel A and treated as described in Figure 2C. Relative qPCR product levels (in %) of the Y1 segment of uORF1 (or its extended variants) recovered from each strain were processed as described in Figure 2C with the values of *TIF32-His uORF1-only* set to 100 (asterisks indicate that *P* < 0.05). (**C**) The YMP1 and YMP28 strains described in Figure 2E were introduced with the uORF4-specific RaP-NiP constructs shown in panel A and treated as described in Figure 2C. Relative qPCR product levels (in %) of the Y4 segment of uORF4 (or its extended variants) recovered from each strain were processed as described in Figure 2C with the values of *TIF32-His uORF4-only* set to 100 (asterisks indicate that *P* < 0.05).

To test this idea with the help of RaP-NiP assay, we took the uORF1- and uORF4-specific RaP-NiP constructs, extended them by two and five alanine codons (Figure 7A), and examined the RNA co-purification in *TIF32* wt *versus Box 6+17* mutant cells. Please note that we did not attempt to create longer extensions due to the aforementioned ‘ribosome occupancy error’. The average ribosomal footprint is generally considered to be around 28–30 nt (49). Thus extending both constructs by 10 codons could mask the effect of the eIF3 gradual dissociation by bringing an extra ribosome to the translated segment Y.

As shown in Figure 7 B and C, three scenarios can be observed. (i) Lengthening of uORF4 by up to 5 Ala codons resulted in progressive reduction of the RNA recovery in both strains (Figure 7C), which undoubtedly reflects the aforementioned effect no. ii (gradual dissociation of eIF3 from elongating ribosomes), because uORF4 does not contain any RPEs. (ii) Extending wt uORF1 by 2 Ala codons showed only a minor drop (by ∼7%) in the RNA recovery in *Box 6+17*, yet it produced the most robust reduction (by ∼33%) in the *TIF32* wt cells (Figure 7B). These differences that are consistent with our hypothesis illuminate the effect no. 1 occurring exclusively in wt cells (displacement of the eIF3-dependent RPEs out of reach of the a/TIF32-NTD) and in principle reveal genetic epistasis between uORF1-2A and *tif32-Box 6+17*. (iii) Further lengthening uORF1 by 5 Ala codons resulted in a similar reduction (by ∼20%) of the RNA recovery in both *TIF32* wt and mutant cells (Figure 7B), which should again reflect mainly the effect no. ii. The latter observations are in accord with our genetic data (21), where we observed that longer uORF1 extensions of 5 or 10 Ala codons were required to detect a significant decrease in β-galactosidase activity in the *TIF32* mutant versus wt strains.

## DISCUSSION

REI is one of the specialized mechanisms of the gene-specific regulation of translation, the occurrence of which is a lot more widespread than it was initially anticipated (7,16,18). The REI efficiency decreases with the increasing length of the uORF (21,50), or if the translation of the uORF is slowed down by strong secondary structures, which causes the translating ribosome to pause (51). These findings, suggesting that the time taken to translate an uORF is more critical than the length of the uORF *per se*, have led to a long-standing theory that some critical initiation factors needed for REI are lost during translation of the uORF rather than during the subunit joining stage of initiation (19). Two lines of evidence further supported but not directly proven this hypothesis. Genetic experiments in budding yeast cells revealed that efficient REI did not occur without intact eIF3 (21,22), and mammalian *in vitro* translation systems with reporter mRNAs containing uORFs required the eIF4F complex to participate in the primary initiation event for efficient REI on the next AUG (20). Based on these observations it was hypothesized that only the 40S ribosome subunits that have kept their contact with eIF3 and eIF4F during elongation until the uORF termination codon are able to resume scanning for the next AUG (reviewed in (2,3)). In addition, Weinberg and colleagues recently observed an existence of what they called the 5΄ ramp of ribosomal footprints that appeared in cells subjected to ribosomal profiling in the absence of the otherwise routine CHX pre-treatment (52). They proposed that elongation might be slower during the early phase of translation perhaps due to an initiation factor that has remained bound to 80S ribosomes during early elongation and maintains the ribosome in a slower state until it stochastically dissociates; their most promising hypothetical candidate for such a factor was indeed eIF3.

Here we provide, to our knowledge, the first direct *in vivo* evidence from yeast cells that, upon subunit joining, eIF3 persistently interacts with the post-initiation 40S subunit for a few elongation cycles and together with the specific REI-promoting features ensures that the post-termination ribosomes are capable to reinitiate downstream. To tackle this problem, it was necessary to develop a new assay that would capture 80S ribosomes bound by initiation factors, in our case by eIF3, while translating short uORFs. We reasoned that the yeast textbook model of REI, the *GCN4* gene preceded by two consecutive uORFs (1 and 2) with high and two (uORFs 3 and 4) with low REI permissiveness, would serve as an ideal model. (i) We showed previously that intact eIF3 was required to allow efficient REI from uORFs 1 and 2 as a function of the genetic interaction between the NTD of the TIF32 subunit of eIF3 and the specific RPEs preceding these two uORFs (22). (ii) We demonstrated that systematic extending of coding regions of uORF1 or uORF4 by up to 10 codons led to gradual drop in REI efficiency that, however, qualitatively differed between these two uORFs and wt *versus* mutant TIF32-NTD (21). By combining the human RIP assay, the CLIP assay, the yeast 43–48S PIC formaldehyde cross-linking assay and the Ni^2+^ affinity chromatography assay (39,42–44), we have established the yeast *in vivo*RNA–Protein Ni^2+^-Pull Down (RaP-NiP) assay (Figure 1), where the scanning PICs, as well as the elongating 80S ribosomes, that are bound by eIF3, are cross-linked, and the specific RNAse H-digested RNA fragments of a defined length are pull down from WCEs by the His-tagged TIF32 subunit of eIF3 and quantified with the help of qPCR. All potential pitfalls of this assay are mentioned in the Results and Materials and Methods sections; here we only want to stress out four key observations lending this novel assay high confidence and specificity. (a) Both REI-permissive uORFs retrieve ∼3–4-fold higher amounts of eIF3-bound RNA fragments compared to the non-permissive ones and mutations in either the RPEs or the NTD of a/TIF32 abolish this difference (Figures 2–5). Hence, we conclude that the stabilizing, REI-promoting interaction between the RPEs and TIF32 does take place and is the major factor behind this difference. (b) Mutating the essential REI-promoting AU-rich motif immediately following the uORF1 stop codon, shown previously to play a dominant role over RPEs (22), also aborted this difference (Figure 6). (c) When the His-tagged eIF2-γ was used as bait, no difference between uORF1 and uORF4 was observed (Figure 3C). This finding rules out that the eIF3-bound scanning PICs that are undoubtedly also captured in our assay significantly contribute to the observed differences. (d) Extending the coding regions of uORFs 1 and 4 led to a gradual drop in the RNA recovery efficiency while preserving the aforementioned difference between uORF1 and uORF4, and confirmed the genetic epistasis between short uORF1 extensions and the mutant a/TIF32-NTD observed before in reporter assays (21). These results strongly suggest that eIF3 stays bound to elongating 80S ribosomes at all *GCN4* uORFs regardless their permissiveness for REI and gradually falls off as the length of the uORF grows. This further underlines the aforementioned conclusion that the major difference in the REI competence are the AU-rich element and RPEs, some of which interact with the a/TIF32-NTD to stabilize the 40S-mRNA-eIF3 pre-reinitiation complex. It also provides a logical explanation for the fold differences observed between uORF1 and uORF4 in RaP-NiP (Figure 2; ∼4-fold) versus β-galactosidase measurements of efficiency of REI (Supplementary Figure S4A; ∼10-fold)—a pure presence of eIF3 at the stop codon of any uORF is simply not enough to make it REI permissive.

Recently, Skabkin *et al*. recapitulated REI *in vitro* on purified, factor-free pre-termination complexes (pre-TCs) assembled on β-globin mRNA derivatives to which a cocktail of preselected eIFs was subsequently added (23). Hence, by definition, this system could not answer the fundamental question of the post-initiation retention of eIF3 and eIF4F on ribosomes translating short uORFs because of the origin of the pre-TCs used in this and other *in vitro* studies (the pre-TCs, formed after translation of short ORFs, were purified by sucrose density gradient centrifugation that strips off all eIFs, which might have remained on 40S subunits during initial cycles of elongation (23)). Nevertheless, the authors showed that if the splitting of post-TCs proceeded in the presence of eIFs 3, 1, 1A and eIF2-TC, 40S subunits remained on mRNA and reinitiated at nearby upstream and downstream AUGs (23). Imposing 3΄-directionality additionally required eIF4F (23). Hence in accord with our previous (21) and current observations, it was concluded that continued association of eIF3 with 40S subunits *in vivo* following translation of short ORFs is essential to promote a reasonable level of downstream reinitiation. Further, it was suggested that requirement for continued association of eIF4F or eIF4G with 40S subunits (20) could be accounted for by the necessity to unwind downstream mRNA secondary structure, if mRNA flanking the stop codon is structured, or by the eIF4G's stabilizing effect on ribosomal association of eIF3 to ensure its retention during translation of short ORFs. The authors also speculated that the inefficient REI *in vivo* after translation of long ORFs could potentially result from potentially low relative concentrations of free eIF3 available to bind pre-TCs *de novo*, in which case, tRNA release from eIF3-unbound 40S subunits would be followed by prompt dissociation of mRNA. However, we have recently showed that eIF3 readily associates with pre-TCs and even controls translation termination and programmed stop codon readthrough *in vivo* in yeast and mammals (53,54). Thus, it is tempting to speculate that it is the lack of eIF4F factors in the pre-TCs, and not of eIF3, that prevents efficient REI after translation of long ORFs. Indeed, we cannot strictly exclude that cells contain an as yet unidentified REI co-factor that is retained with eIF3 during early elongation cycles but is not dragged by eIF3 to the pre-TC. In any case, it will be intriguing to employ the RaP-NiP assay in the follow-up study and examine whether the eIF4F factors are also required for efficient REI in yeast and remain bound to elongating 80S ribosomes post-initiation or not.

Bearing in mind the highly variable transcript-specific structural properties of various uORFs, the unequivocal regulatory potential of uORFs, and the fact that uORF mutations may be involved in the genetic architecture of a wide variety of diseases, it is out of question that a precise definition of all factors ensuring REI competence, as well as understanding the molecular mechanism of REI in detail, represent important challenges for future research. Developing new tools such as the one presented here will certainly help us to stand up to these challenges. Besides, we believed the RaP-NiP will also find its use in studies of *in vivo* requirements and mechanics of ribosomal scanning from the 5΄ end of mRNA to the AUG start codon and the role of eIF3 and other factors in canonical translation termination and programmed readthrough.

## Supplementary Material

Supplementary Data
