# Supplementary material for: In vivo evidence that eIF3 stays bound to ribosomes elongating and terminating on short upstream ORFs to promote reinitiation
Source: Nucleic Acids Res. 2017 Jan 24;45(5):2658–74. doi: 10.1093/nar/gkx049 (PMC5389480; doi:10.1093/nar/gkx049)
Supplement: Supplementary Data [file gkx049_supplementary_data.zip › nar-02906-a-2016-File009.pdf]

SUPPLEMENTARY MATERIAL

SUPPLEMENTARY MATERIALS AND METHODS

Construction of plasmids and yeast strains

Strain YVM1 carrying chromosomal deletion of *GCN4* gene together with its 5´ UTR was constructed in the following steps. (i) Primers VM4 (it is a composite primer, the 5' part of which base pairs with the sequence corresponding to the extreme 5' UTR of the *GCN4* gene [far upstream of all RPEs] and the discontinuous 3' part of which base pairs with the sequence corresponding to the very beginning of the *GCN4::KanMx* cassette) and VM25 (base pairing with the very end of the *GCN4::KanMx* cassette) were used to PCR amplify a DNA fragment containing the *GCN4::KanMx* cassette using the genomic DNA isolated from the Euroscarf strain BY4741 *gcn4Δ* as the template. (ii) The purified PCR product was then used to transform YBS52 to delete the *GCN4* gene and its entire 5' UTR and the resulting transformants were selected for the G418 resistance. The deletion of *GCN4* and its 5' UTR was verified by PCR.

To generate the yeast strains YMP1, YMP6, YMP9, YMP10, YMP28, YMP34, and YMP77, YVM1 was first transformed with plasmids p196, pMP11, p680, pMP66, pSG42, pMP58 and p199, respectively, and the uracil auxotrophy was regained by growing the cells that have evicted the original pRS-*a/TIF32-His-URA* on SD plates containing 5-fluoroorotic acid.

All plasmids listed below were created with the help of PCR using the indicated pairs of primers and DNA templates. For pMP29: VM1 / MP43 and p180 as template; for pMP34: MP43 / MP5 and pSG60; for pMP36: MP43 / MP5 and pSG138; for pMP37: MP43 / MP5 and pSG141; for pMP47: MP43 / MP5 and pSG93; for pMP56: MP43 / MP138 and p209; for pMP57: MP43 / MP144 and p209; pMP59: MP43 / MP161 and p558; pMP60: MP43 / MP161 and p559; pMP62: MP43 / MP162 and pMP30; pMP63: MP43 / MP163 and pMP30. The resulting PCR products were digested with *Sal*I and *Bst*EII and ligated into *Sal*I-*Bst*EII cleaved p180 .

The plasmids described below were produced with the help of fusion PCR using the indicated two pairs of primers and DNA templates: for pMP30 (i) MP91 / MP93 and p226 as template and (ii) VM52 / MP43 and p226; for pMP32 (i) VM72 / MP43 and p209 and (ii) VM73 / VM1 and p209; for pMP33 (i) VM40 / MP43 and p209 and (ii) VM38 / VM1 and p209; for pMP35 (i) MP91 / MP92 and p227 and (ii) VM52 / MP43 and p227; and for pMP53 (i) MP130 / MP43 and pSG61 and (ii) MP129 / MP46 and pSG61, respectively. Thus obtained PCR products were used in a 1:1 ratio as templates for a third PCR amplification with primers MP43 / MP91 (for pMP30 and pMP35), MP43 / VM1 (for pMP32 and pMP33), MP43 / MP46 (for pMP53). The resulting PCR products were cleaved with *Sal*I and *Bst*EII and ligated into equally digested p180.

pMP58 was created by inserting the *PstI-SacI* digested pMP11 into p355 digested with the same enzymes.

SUPPLEMENTARY REFERENCES

1. Gunisova, S. and Valasek, L.S. (2014) Fail-safe mechanism of GCN4 translational control-uORF2 promotes reinitiation by analogous mechanism to uORF1 and thus secures its key role in GCN4 expression. *Nucleic Acids Res*, **42**, 5880-5893.
2. Gunisova, S., Beznoskova, P., Mohammad, M.P., Vlckova, V. and Valasek, L.S. (2016) In-depth analysis of cis-determinants that either promote or inhibit reinitiation on GCN4 mRNA after translation of its four short uORFs. *RNA*, **22**, 542-558.
3. Valášek, L., Nielsen, K.H. and Hinnebusch, A.G. (2002) Direct eIF2-eIF3 contact in the multifactor complex is important for translation initiation in vivo. *EMBO J*, **21**, 5886-5898.
4. Munzarová, V., Pánek, J., Gunišová, S., Dányi, I., Szamecz, B. and Valášek, L.S. (2011) Translation Reinitiation Relies on the Interaction between eIF3a/TIF32 and Progressively Folded cis-Acting mRNA Elements Preceding Short uORFs. *PLoS Genet*, **7**, e1002137.
5. Mueller, P.P. and Hinnebusch, A.G. (1986) Multiple upstream AUG codons mediate translational control of *GCN4*. *Cell*, **45**, 201-207.
6. Grant, C.M. and Hinnebusch, A.G. (1994) Effect of sequence context at stop codons on efficiency of reinitiation in *GCN4* translational control. *Mol Cell Biol*, **14**, 606-618.
7. Mueller, P.P., Harashima, S. and Hinnebusch, A.G. (1987) A segment of *GCN4* mRNA containing the upstream AUG codons confers translational control upon a heterologous yeast transcript. *Proc Natl Acad Sci USA*, **84**, 2863-2867.
8. Gietz, R.D. and Sugino, A. (1988) New yeast-Escherichia coli shuttle vectors constructed with in vitro mutagenized yeast genes lacking six-base pair restriction sites. *Gene*, **74**, 527-534.
9. Sikorski, R.S. and Hieter, P. (1989) A system of shuttle vectors and yeast host strains designed for efficient manipulation of DNA in *Saccharomyces cerevisiae*. *Genetics*, **122**, 19-27.
10. Szamecz, B., Rutkai, E., Cuchalova, L., Munzarova, V., Herrmannova, A., Nielsen, K.H., Burela, L., Hinnebusch, A.G. and Valášek, L. (2008) eIF3a cooperates with sequences 5' of uORF1 to promote resumption of scanning by post-termination ribosomes for reinitiation on GCN4 mRNA. *Genes Dev*, **22**, 2414-2425.
11. Chiu, W.-L., Wagner, S., Herrmannová, A., Burela, L., Zhang, F., Saini, A.K., Valášek, L. and Hinnebusch, A.G. (2010) The C-Terminal Region of Eukaryotic Translation Initiation Factor 3a (eIF3a) Promotes mRNA Recruitment, Scanning, and, Together with eIF3j and the eIF3b RNA Recognition Motif, Selection of AUG Start Codons. *Mol Cell Biol*, **30**, 4415-4434.
12. Khoshnevis, S., Gunišová, S., Vlčková, V., Kouba, T., Neumann, P., Beznosková, P., Ficner, R. and Valášek, L.S. (2014) Structural integrity of the PCI domain of eIF3a/TIF32 is required for mRNA recruitment to the 43S pre-initiation complexes. *Nucleic Acids Research*, **42**, 4123-4139.
13. Gomez, E., Mohammad, S.S. and Pavitt, G.D. (2002) Characterization of the minimal catalytic domain within eIF2B: the guanine-nucleotide exchange factor for translation initiation. *Embo J*, 5292-5301.

SUPPLEMENTARY FIGURE LEGENDS

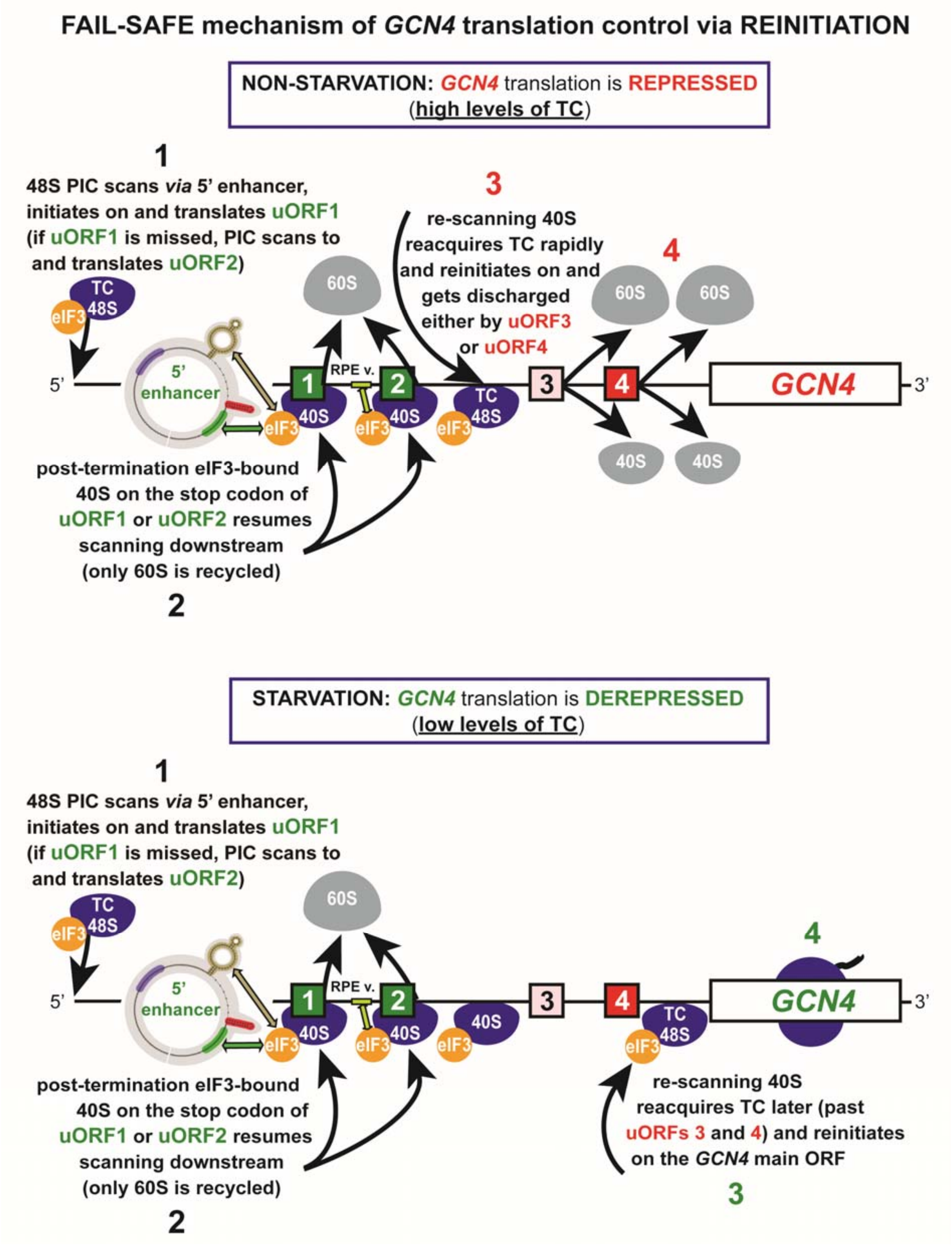

Supplementary Fig. 1. Model of the 'Fail-safe mechanism' of *GCN4* translational control. Schematic of the *GCN4* mRNA leader showing the distribution of all four short uORFs, locations of the uORF1-specific and uORF2-specific RPEs, 40S-bound eIF3, and the description of the 'Fail-safe mechanism' of the *GCN4* translational control -

adapted from (1). Upper panel models the events on the *GCN4* mRNA leader occurring under non-starvation conditions with abundant TC levels – '*GCN4*-expression repressed state', the lower panel illustrates the steps that take place under starvation condition with a limited supply of the TC – '*GCN4*-expression derepressed state' (see the main text for further details).

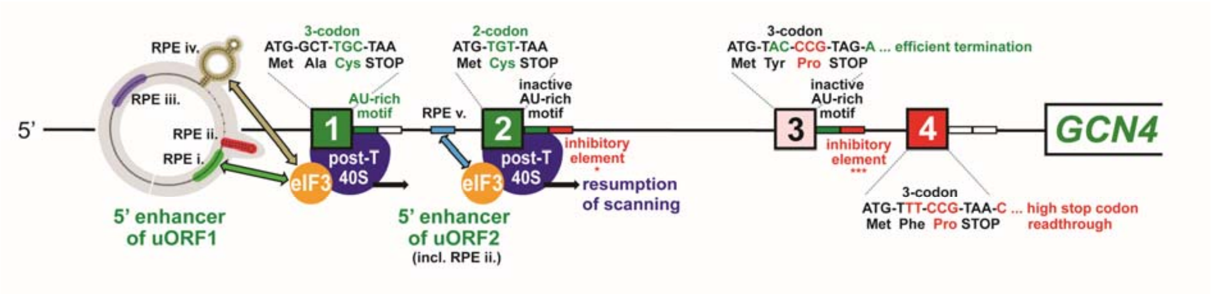

Supplementary Fig. 2. Summary of all *cis*-determinants that either promote or inhibit reinitiation on *GCN4* after translation of its four short uORFs. Schematics of the 5' enhancers of uORF1 and 2 containing their respective RPEs, some of which functionally interact with eIF3 to promote the resumption of scanning – adapted from (2). Green color-coding generally indicates stimulatory effects of the corresponding *cis*-factors on efficiency of REI, whereas red color-coding indicates inhibitory effects (with the exception of RPE ii. of uORF1 which is also stimulatory); the number of asterisks below the inhibitory elements of the uORF2 and uORF3 3' sequences illustrates the degree of their inhibition (see the main text for further details).

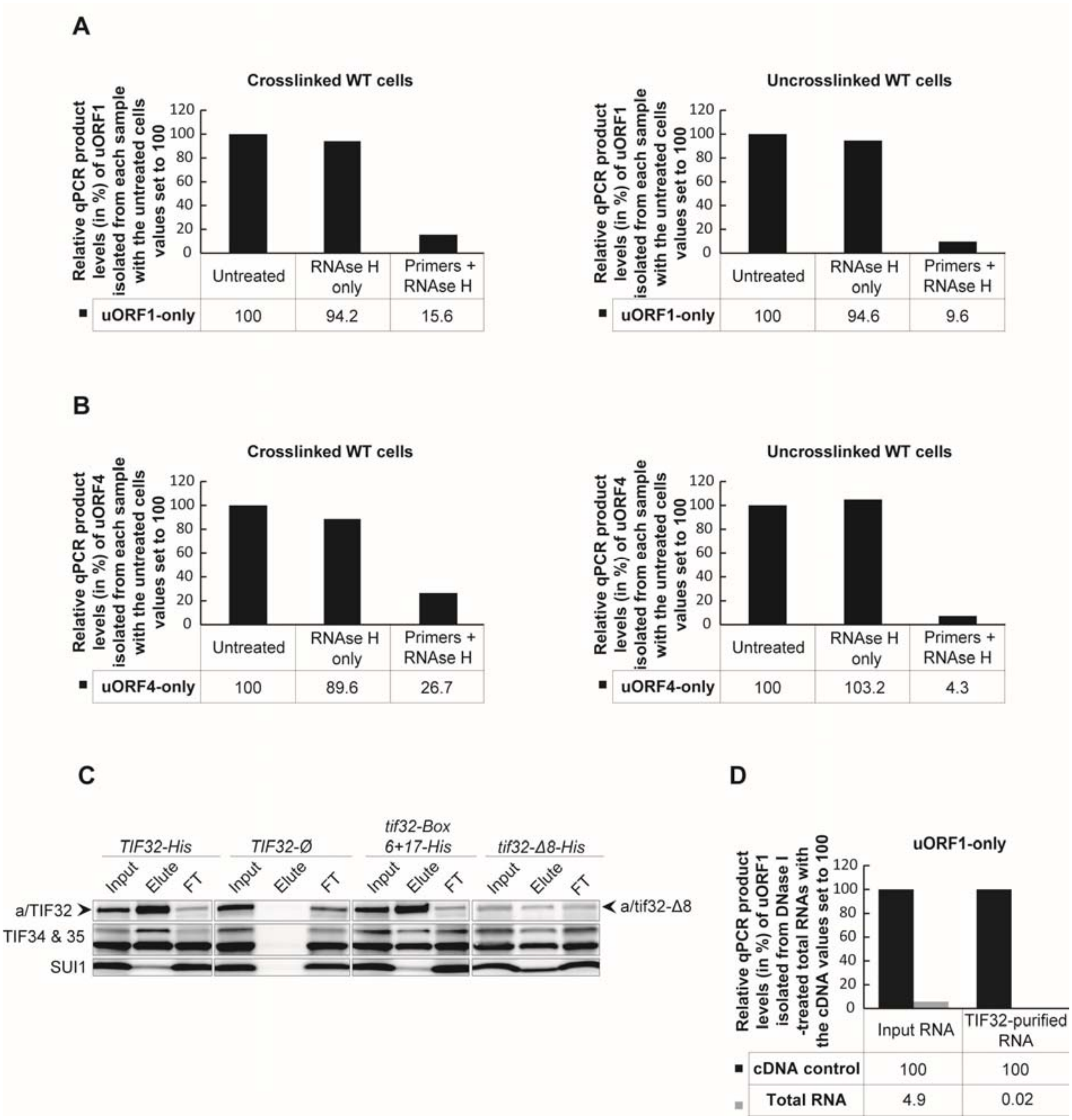

Supplementary Fig. 3. **The efficiency and specificity of the RNAse H digestion and of the Ni^{2+} affinity chromatography.** (A) The YMP1 (*gcn4Δ TIF32-His*) strain was introduced with the uORF1-only RaP-NiP construct shown in Fig. 2B and the WCEs from formaldehyde crosslinked (left panel), as well as uncrosslinked wt (right panel) cells, were examined for efficiency and specificity of RNAse H digestion using sequence-specific custom made primers (indicated by scissors in Fig. 2A). Both cell cultures were either untreated or incubated with either only RNase H or RNase H plus the primers. The efficiency and specificity of the RNase H digestion were monitored by qRT-PCR with two specific primers, one of which base-pairs with the sequence immediately preceding the 5' RNase H cleavage site and the other base-pairs with the sequence immediately following the 3' RNase H cleavage site. This way only undigested mRNAs could give rise to specific qRT-PCR products. Relative qPCR

product levels (in %) of the Y1 segment of uORF1 isolated from the crosslinked or uncrosslinked strain by hot phenol extraction were processed as described in Fig. 2C with the values of untreated cells set to 100%. (B) The YMP1 (*gcn4Δ TIF32-His*) strain was introduced with the uORF4-only RaP-NiP construct shown in Fig. 2B and the WCEs from formaldehyde crosslinked (left panel), as well as uncrosslinked wt (right panel) cells, were examined for efficiency and specificity of RNAse H digestion as described in panel A. Relative qPCR product levels (in %) of the Y4 segment of uORF4 isolated from the crosslinked or uncrosslinked strain by hot phenol extraction were processed as described in Fig. 2C with the values of untreated cells set to 100%. (C) Western blotting of the Ni^{2+} affinity chromatography performed with the YMP1, YMP28, and YMP77 strains showing 2 subunits of eIF3 and eIF1/SUI1 co-immunoprecipitating with the His-tagged *a*/TIF32 subunit of eIF3 as described before (3). The *a*/TIF32 untagged strain (TIF32-*Ø*) was used as a negative control. (D) The YMP1 (*gcn4Δ TIF32-His*) strain was introduced with the uORF1-only RaP-NiP construct shown in Fig. 2B and total RNA isolated by hot phenol extraction was analyzed for DNA contamination after DNAse I treatment before (Input RNA) and after (TIF32-purified RNA) the Ni^{2+} affinity chromatography. cDNAs generated by RT-PCR from the same total RNA preparations were used as reference controls (100 %). Y1 segments of uORF1 isolated from the Dnase I-treated total RNA samples before or after Ni^{2+} pull down were processed as described in Fig. 2C with the values of cDNA controls set to 100%.

Supplementary Figure 4

A

	a/TIF32	a/tif32-Box-6+17	a/tif32-Δ8
**uORF1-only**	467.85 ± 53.4<br>**100%**	168.15 ± 67.18<br>**35.9%**	101.38 ± 2.8<br>**21.6%**
**uORF4-only**	49.59 ± 2.1<br>**10.6%/100%**	45.37 ± 1.9<br>**91.5%**	37.54 ± 2.1<br>**75.7%**
**uORF3-only**	178.78 ± 15.3<br>**38.2%**		
**uORF2-only**	767.37 ± 21.7<br>**100%**	306.92 ± 82.1<br>**40%**	331.08 ± 27.9<br>**43%**
**uORF4_2-only**	56.85 ± 18.0<br>**7.4%**		

Units of β-galactosidase activity
Activity relative to a/TIF32 uORF1-only or uORF-2 only construct

B

	a/TIF32
**uORF1-only**	800.16 ± 45.95<br>**100%**
**uORF1-SUB40**	546.14 ± 41.56<br>**68.25%**
**uORF1-CAAII**	490.21 ± 89.99<br>**61.26%**
**uORF1-1114**	257.11 ± 27.25<br>**32.13%**
**uORF1-1144**	123.9 ± 25.99<br>**15.48%**
**uORF1-2A**	626.38 ± 74.08<br>**78.28%**
**uORF1-5A**	381.26 ± 10.06<br>**47.64%**

Units of β-galactosidase activity
Activity relative to uORF1-only construct

C

	a/TIF32
**uORF2-only**	1304.2 ± 155.8<br>**100%**
**uORF2-SUB18**	761.73 ± 56.53<br>**58.40%**
**uORF2-CAAII & SUB18**	268.48 ± 30.03<br>**20.58%**

Units of β-galactosidase activity
Activity relative to uORF2-only construct

D

	a/TIF32
**uORF4-only**	89.53 ± 8.85<br>**100%**
**uORF4-2A**	72.57 ± 11.39<br>**81.05%**
**uORF4-5A**	53.44 ± 6.52<br>**59.68%**

Units of β-galactosidase activity
Activity relative to uORF4-only construct

Supplementary Figure 4. The Rap-Nip constructs show expected β-galactosidase activities reflecting REI-permissive character of uORF1 and 2, dependent on their REI-promoting elements, and REI-non-permissive character of uORF3 and 4. (A) The H567 (Ycp-*TIF32*), H573 (Ycp-tif32-*Box-6+17*) and H568 (*Ycp-tif32-Δ8*) strains were introduced with the uORF-only *GCN4-lacZ* constructs shown in Figure 2B. The resulting transformants were pre-cultured in minimal media overnight, diluted to

OD_{600}~0.35, grown for additional 6 h and the β-galactosidase activities were measured in the WCEs and expressed in units of nmol of *o*-nitrophenyl-β-d-galactopyranoside hydrolyzed/min/mg of protein. The mean values and standard deviations obtained from at least three independent measurements with three independent transformants, and activities of the respective constructs relative to a/TIF32 uORF1-only or uORF2-only are given in the table. (B) The H567 (Ycp-*TIF32*) strain was introduced with the uORF1-specific *GCN4-lacZ* constructs shown in Figure 4A, 6A and 7A and treated as described in panel A. The mean values and standard deviations obtained from at least three independent measurements with three independent transformants, and activities of the respective constructs relative to a/TIF32 uORF1-only are given in the table. (C) The H567 (Ycp-*TIF32*) strain was introduced with the uORF2-specific *GCN4-lacZ* constructs shown in Figure 5A and treated as described in panel A. The mean values and standard deviations obtained from at least three independent measurements with three independent transformants, and activities of the respective constructs relative to a/TIF32 uORF2-only are given in the table. (D) The H567 (Ycp-*TIF32-His*) strain was introduced with the uORF4-specific *GCN4-lacZ* constructs shown in Figure 7A and treated as described in panel A. The mean values and standard deviations obtained from at least three independent measurements with three independent transformants, and activities of the respective constructs relative to a/TIF32 uORF4-only are given in the table.

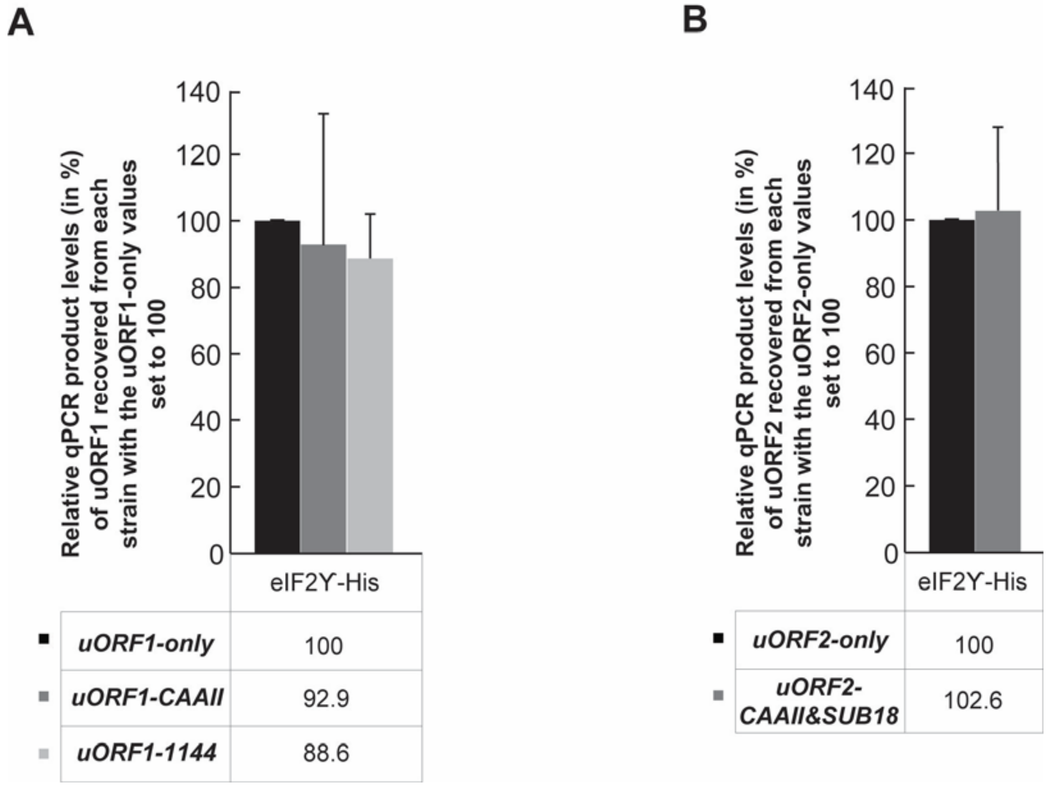

Supplementary Figure 5. eIF2 shows no preference in association with RNA segments encompassing.either wt or mutant uORF1 (A) or uORF2 (B). The YMP34 (*gcn4Δ TIF32)* strain was introduced with the (A) uORF1-specific or (B) uORF2-specific RaP-NiP constructs shown in Fig. 4A, 5A and 6A along with pMP65, carrying all three subunits of eIF2 with *GCD11* allele bearing a His tag, and treated as described in Fig. 2C. Relative qPCR product levels (in %) of the corresponding Y1 or Y2 segments of uORF1 or uORF2 recovered from each strain were processed as described in Fig. 2C with the values of *GCD11-His* uORF1-only or uORF2-only set to 100.

SUPPLEMENTARY TABLES

Supplementary Table 1. Yeast strains used in this study.

Strain	Genotype	Source or reference
BY4741 *gcn4Δ*	*MATa gcn4::KanMX4 his3Δ1 leu2Δ0 met15Δ0 ura3Δ0*	Euroscarf
YBS52	*MATa leu2-3, -112 ura3-52 trp1Δ gcn2Δ tif32Δ URA3::GCN2 ura3* (*pRS-a/TIF32-His-URA*)	(4)
YVM1	*MATa gcn4::KanMX (incl. 5' UTR), leu2-3, -112 ura3-52 trp1Δ gcn2Δ tif32Δ URA3::GCN2 ura3* (*pRS-a/TIF32-His-URA*)	this study
YMP1	*MATa gcn4::KanMX (incl. 5' UTR), leu2-3, -112 ura3-52 trp1Δ gcn2Δ tif32Δ URA3::GCN2 ura3* (*pRS-a/TIF32-His-LEU*)	this study
YMP6	*MATa gcn4::KanMX (incl. 5' UTR), leu2-3, -112 ura3-52 trp1Δ gcn2Δ tif32Δ URA3::GCN2 ura3* (*pRS-a/TIF32-LEU*)	this study
YMP9	*MATa gcn4::KanMX (incl. 5' UTR), leu2-3, -112 ura3-52 trp1Δ gcn2Δ tif32Δ URA3::GCN2 ura3* (*pRS-a/tif32R731I-His-LEU*)	this study
YMP10	*MATa gcn4::KanMX (incl. 5' UTR), leu2-3, -112 ura3-52 trp1Δ gcn2Δ tif32Δ URA3::GCN2 ura3* (*pRS-a/tif32-Box34-His-LEU*)	this study
YMP28	*MATa gcn4::KanMX (incl. 5' UTR), leu2-3, -112 ura3-52 trp1Δ gcn2Δ tif32Δ URA3::GCN2 ura3* (*pRS-a/tif32-Box6+17-His-LEU*)	this study
YMP77	*MATa gcn4::KanMX (incl. 5' UTR), leu2-3, -112 ura3-52 trp1Δ gcn2Δ tif32Δ URA3::GCN2 ura3* (*pRS- a/tif32-Δ8-His-LEU*)	this study
YMP34	*MATa gcn4::KanMX (incl. 5'UTR), leu2-3, -112 ura3-52 trp1Δ gcn2Δ tif32Δ URA3::GCN2 ura3 (pRS-a/TIF32-TRP)*	this study
H567	*MATa leu2-3, -112 ura3-52 trp1Δ gcn2Δ tif32Δ URA3::GCN2 ura3 (YCp-TIF32-LEU)*	(4)
H568	*MATa leu2-3, -112 ura3-52 trp1Δ gcn2Δ tif32Δ URA3::GCN2 ura3 (YCp-a/tif32-Δ8-LEU)*	(4)
H573	*MATa leu2-3, -112 ura3-52 trp1Δ gcn2Δ tif32Δ URA3::GCN2 ura3 (YCp-a/tif32-Box6+17-LEU)*	(4)

Supplementary Table 2. Plasmids used in this study.

Plasmid	Description	Source or reference
p180 (*YCp50–GCN4–lacZ*)	low copy *URA3* vector containing WT *GCN4* leader fused with *lacZ*	(5)
p209 (uORF1-only)	low copy *URA3* vector containing uORF1 only at its original position	(6)
p226 (uORF4-only)	low copy *URA3* vector containing uORF4 only at its original position	(7)
p227 (uORFless)	low copy *URA3* vector containing uORFless *GCN4* leader created by point mutations in uORF1 (*Hind*III), uORF2 (*Eco*RI), uORF3 (*Kpn*I) and uORF4 (*Bgl*II)	(7)
p353 (*YCplac22-URA3*)	low copy *URA3* empty vector	(8)
p355 (*pRS314*)	low copy *TRP1* empty vector	(9)
p558 (uORF1-2A)	low copy *URA3* vector containing uORF1 only at its original position; uORF1 contains 2 Ala codons insertion	(10)
p559 (uORF1-5A)	low copy *URA3* vector containing uORF1 only at its original position; uORF1 contains 5 Ala codons insertion	(10)
pSG60 (uORF2-only)	low copy *URA3* vector containing uORF2 only at its original position	(1)
pSG61 (uORF3-only)	low copy *URA3* vector containing uORF3 only at its original position	(1)
pSG93 (uORF2-CAAII)	low copy *URA3* vector containing uORF2 only at its original position; the sequences -76 to -55 upstream of uORF1 were substituted by a stretch of CAA triplets of the identical length to the original sequence	(1)
pSG138 (uORF2-only-SUB18)	low copy *URA3* vector containing uORF2 only at its original position; the sequences +41 to +50 upstream of uORF2 were substituted by complementary sequences	(1)
pSG141 (uORF2-CAAII-SUB18)	low copy *URA3* vector containing uORF2 only; the mutations CAAII and SUB18 were combined	(1)
pMP29 (uORF1-only)	low copy *URA3* vector containing uORF1 only with its genuine 5' and 3' flanking sequences	this study
pMP30 (uORF4-only)	low copy *URA3* vector containing uORF4 only with its genuine 5' and 3' flanking sequences	this study

pMP32 (uORF1-SUB40)	low copy *URA3* vector containing uORF1 only; the sequences -48 to -40 upstream of uORF1 were substituted by complementary sequences	this study
pMP33 (uORF1-CAAII)	low copy *URA3* vector containing uORF1 only; the -143 to -122 upstream sequences were substituted by a stretch of CAA triplets of the identical length to the original sequence	this study
pMP34 (uORF2-only)	low copy *URA3* vector containing uORF2 only with its genuine 5’ and 3’ flanking sequences	this study
pMP35 (uORF4_2-only)	low copy *URA3* vector containing uORF4 only with its genuine 5’ and 3’ flanking sequences	this study
pMP36 (uORF2-SUB18)	low copy *URA3* vector containing uORF2 only; the sequences +41 to +50 upstream of uORF2 were substituted by complementary sequences	this study
pMP37 (uORF2-CAAII&SUB18)	low copy *URA3* vector containing uORF2 only; the mutations CAAII and SUB18 were combined	this study
pMP47 (uORF2-CAAII)	low copy *URA3* vector containing uORF2 only; the -143 to -122 upstream sequences were substituted by a stretch of CAA triplets of the identical length to the original sequence	this study
pMP53 (uORF3-only)	low copy *URA3* vector containing uORF3 only with its genuine 5’ and 3’ flanking sequences	this study
pMP56 (uORF1-1114)	low copy *URA3* vector containing uORF1 only; the 3´ flanking sequence (25 nt beyond the stop codon) was replaced by the corresponding sequence of uORF4	this study
pMP57 (uORF1-1144)	low copy *URA3* vector containing uORF1 only; the coding sequence and the 3´ flanking sequence (25 nt beyond the stop codon) were replaced by the corresponding sequences of uORF4	this study
pMP59 (uORF1-2A)	low copy *URA3* vector containing uORF1 only coding sequence extended by 2 Alanine residues (MAA_2C) in pMP29	this study

pMP60 (uORF1-5A)	low copy *URA3* vector containing uORF1 only coding sequence extended by 5 Alanine residues (MAA_5C) in pMP29	this study
pMP62 (uORF4-2A)	low copy *URA3* vector containing uORF4 only coding sequence extended by 2 Alanine residues (MFA_2P) in pMP30	this study
pMP63 (uORF4-5A)	low copy *URA3* vector containing uORF4 only coding sequence extended by 5 Alanine residues (MFA_5P) in pMP30	this study
p196 (*pRS-a/TIF32-His-LEU*)	single copy *TIF32-His* in *LEU2* plasmid, from pRS315	(3)
p680 (*pRS-a/tif32R731I-His-LEU*)	low-copy *tif32-R731I-His* in *LEU2* plasmid, from pRS315	(11)
pMP66 (*pRS-a/tif32-Box34-His-LEU*)	low-copy *tif32-Box34-His* in *LEU2* plasmid, from pRS315	(12)
pMP11 (*pRS-a/TIF32-LEU*)	low-copy *TIF32* in *LEU2* plasmid, from pRS315	this study
pMP58 (*pRS314-a/TIF32-TRP*)	low-copy *TIF32* in *TRP1* plasmid, from pRS314	this study
pSG42 (*pRS-a/tif32-Box6+17-His-LEU*)	low-copy *tif32-Box6+17-His* in *LEU2* plasmid, from pRS315	this study
p199 (*pRS-a/tif32-Δ8-His-LEU*)	low-copy *tif32-Δ8-His* in *LEU2* plasmid, from pRS315	this study
pMP65 (*pRS-GCD11-His-LEU*)	low-copy *GCD11-His, SUI2, SUI3* in *LEU2* plasmid, from pRS425	(13)

Supplementary Table S3. Oligonucleotides used in this study.

Oligonucleotide	Sequence (5' to 3')
VM1	TTTCTAATAAGGTAACCACTTTAAAAACAAAATATAATCGGTTTAGCAAGCCATTTTTCAATGATCTTTAAT TTTTTAATACGATACTGATAATAACTT
VM4	TCGGCTCGCTGTCTTACCTTTTAAAATCTTCTACTTCTTGACAGTACTTATCTTCTTATATAATAGATATCG TACGCTGCAGGTCGAC
VM25	TTTAAAGTTTCATTCCAGCATTAGC
VM38	CAGTTTATTAAGTTATTATCAGTA
VM40	TACTGATAATAACTTAATAAACTGATTGTTGTTGTTGTTGTTGTTGTTGATTGCGAAGTAGATGAGTGAG
VM52	GTAGATGAGTGAGCTGTGTGGCTGG
VM72	ATTTTTTAATACGATACTGATAATTTGAATTATAACTGAACTAAAATAAAATATTTTG
VM73	ATTATCAGTATCGTATTAAAAAAT
MP5	CCTGGATAATTTGACAGAAAGGTAACCAAATAAATTTTCTCTTTCAATAAATTTAACACATAATT
MP7	GGGAAATTTTTATTGGCGAGTAAAC
MP8	GCTCACTCATCTACTTCGCAATC
MP43	GTTGAAGGCTCTCAAGGGCATC
MP46	TGGATAATTTGACAGAAAGGTAACC

MP79	GTGGCTGGTGAGTTGTATAATTCGC
MP91	AAGGTAACCAAATTCTTGAATAAAATTGTAACCGTTACGGAAACATCTTGAATAAAATTC
MP92	CCAGCCACACAGCTCACTCATCTACTTCGCAATCCTGTGTTAAATTTATTGAAAGAGAAA
MP93	CCAGCCACACAGCTCACTCATCTACTTCGCAATCTTGAAAACTGTCAGTTTTTTGAAGAG
MP129	ATTAATTAAAGTCCTTTACTTTTTTTG
MP130	CAAAAAAAGTAAAGGACTTTAATTAATGATTGCGAAGTAGATGAGTGAGC
MP138	GAAAGGTAACCAAATTCTTGAATAAAATTGTAACCGTTAGCAAGCCATTTTTCAATGATC
MP143	GCA GAC AAA TTG GTA AAC AAA AC
MP144	GAAAGGTAACCAAATTCTTGAATAAAATTGTAACCGTTACGGAAACATTTTTCAATGATCTTTAATTTTTTA ATAC
MP145	GCCTTCTACGTTTCCATCCA
MP146	GGCCAAATCGATTCTCAAAA
MP161	GAA AGG TAA CCA CTT TAA AAA CAA AAT ATA ATC GG
MP162	GAA AGG TAA CCA AAT TCT TGA ATA AAA TTG TAA CCG TTA CGG AGC AGC AAA CAT CTT GAA TAA AAT TCT ACG G
MP163	GAA AGG TAA CCA AAT TCT TGA ATA AAA TTG TAA CCG TTA CGG AGC AGC AGC AGC AGC AAA CAT CTT GAA TAA AAT TCT ACG G
MP178	TTTTTTTTGCCCATCAGTTTCACTAG
